# Optimal multi-objective energy management of decentralized demand response incorporating uncertainties

**DOI:** 10.1371/journal.pone.0328838

**Published:** 2025-07-28

**Authors:** Alireza Norouzpour Shahrbejari, Mohammad Hadi Eskandari Sani, Mehdi Zareian Jahromi, Elnaz Yaghoubi, Elaheh Yaghoubi, Mohammad Reza Maghami

**Affiliations:** 1 Department of Electrical Engineering, Technical and Vocational University (TVU), Tehran, Iran; 2 Department of Electrical Engineering, Amirkabir University of Technology, Tehran, Iran; 3 Department of Electrical and Electronics Engineering, Karabuk University, Karabuk, Turkey; 4 Strategic Research Institute (SRI), Asia Pacific University of Technology and Innovation (APU), Kuala Lumpur, Malaysia; Memorial Sloan Kettering Cancer Center, UNITED STATES OF AMERICA

## Abstract

This paper presents a decentralized demand response (DR) framework that, incorporating optimal multi-objective energy management strategies, addresses uncertainties in power networks. The power industry faces challenges in operational optimization due to uncertainties in generation and consumption while evaluating environmental impacts and long-term economic implications. This research introduces an innovative approach by combining advanced DR techniques with a robust energy management strategy designed for uncertain conditions, enhanced by sensitivity analysis to key system parameters. The article considers a network with distributed generating resources, including wind turbines, microturbines, photovoltaics, energy storage systems (ESS), and diesel generators, where generation is controlled hourly based on load fluctuations. Energy consumption optimization requires not only distributed energy generation but also DR to variations in demand, ensuring system reliability under diverse scenarios. Consumers play a crucial role in optimizing energy usage through incentive-based participation. To achieve the research goal of reducing generation and purchasing costs in power grids through optimal energy management and DR to fluctuations, a stochastic approach is employed to obtain the best outcomes. This paper proposes a novel method for optimizing energy consumption in power networks by integrating stochastic techniques to manage uncertainties and variable conditions. The findings show improved network efficiency and cost reduction, achieving a 15.62% decrease in voltage deviation, 37.08% reduction in load demand, 62.05% decrease in active losses, 81.25% reduction in reactive losses, and 33–45% reduction in Expected Energy Not Supplied (EENS).

## 1. Introduction

The ever-expanding industrialization and the increasing demands on distribution networks have intensified challenges for grid planners and administrators. These challenges include significant power losses and unexpected voltage fluctuations, imposing considerable financial burdens on utility companies [1,2]. The modern power grid, especially those integrated with renewable energy sources (RESs), faces critical issues in managing the inherent volatility of power generation and consumption while also addressing environmental and long-term economic impacts [3]. RES, such as wind and solar, introduce operational variability that complicates grid management, increases reliance on diesel generators during shortages, raising concerns about carbon emissions and challenges voltage stability [4–6]. The industrial, residential, and commercial sectors are considered in this study. While uncertainties related to renewables integration were primarily handled in previous studies [7], this research advanced those efforts by integrating an adaptive energy management system (EMS) with DR programs and incorporating energy storage systems (ESS). Through the integration of these components, renewable energy fluctuations were reduced, grid operations were stabilized, system reliability was enhanced, and consumer costs were minimized through incentive and penalty schemes. The proposed framework enables simultaneous optimization of energy generation and consumption, addressing the intrinsic uncertainties of modern power systems [8]. As decentralized power systems become more complex, centralized planning remains crucial for effective management [9]. This study emphasizes the need for flexibility in EMS and DR systems to counteract resource and renewable energy fluctuations.

### 1.1. Literature review

The approaches reviewed in the literature are widely recognized as effective methods for optimizing energy use while ensuring economic and technical efficiency to meet the consumer demand. Although some techniques have been established as standard for improving MG performance, uncertainty management has gained increased attention from both the scientific and industrial sectors. Various methods have been proposed to model uncertainty in conventional distribution systems and MGs, including experimental techniques, probabilistic approaches, literature-based reviews [10], and membership-based modeling methods [11–13]. These approaches rely on natural systems where the input variables are difficult to predict. Variability remains the most common source of uncertainty, while membership functions are typically developed based on prior experience. Scenario generation techniques help define control probabilities [14] and consider worst-case scenarios [15] within MGs, helping to mitigate the inherent intermittency of RES. With increasing research into hybrid EMS for improving MG performance, the integration of DR programs into MG operations has also gained interest [16]. Despite the potential benefits of collaboration between DR programs, DG units powered by RES, and shared ESSs, little research has thoroughly examined the integration of DR with EVs [17,18].

A substantial body of research on MG energy management and optimization focuses on reducing operational costs, improving voltage stability, and optimizing DG resource utilization. Many studies proposed multi-objective optimization techniques to enhance system performance. However, challenges related to uncertainty in renewable energy generation and its interaction with DR programs remain insufficiently addressed. For instance, study [19] applies an improved quantum-behaved particle swarm optimization (QPSO) algorithm to solve short-term economic-environmental optimization problems in MGs, incorporating wind turbines (WT), photovoltaic (PV) panels, and fuel cells. While the results show improved performance, the study does not comprehensively address uncertainties in renewable energy generation or the effects of DR programs. Similarly, study [20] utilizes the improved decomposition-based evolutionary algorithm (I-DBEA) for optimizing DG placement, minimizing power losses, and reducing VD, but does not consider the combined effects of DR programs and renewable resources on system-wide performance.

Other studies have explored load scheduling optimization within MGs. For example, an alternating current optimal power flow (AC-OPF) problem has been proposed to enhance real-time load scheduling through competitive algorithms. Most research on multi-energy systems (MES), such as study [21], has focused on optimizing energy exchange between diverse sources and assessment system behavior under uncertainty. Although these studies highlight economic and environmental benefits, they fail to address the role of DR programs and their impact on costs and system performance is not represented. In [22], a two-stage stochastic optimization framework is introduced for real-time MG operation, considering flexible resources such as responsive loads and fast-acting generators. Study [23] explores the potential benefits of hydrogen storage and DR programs in MG performance optimization, illustrating that hydrogen storage can significantly reduce operational costs. However, further research is required to assess its interaction with other DGs and DR programs. Despite extensive research on MG optimization and energy management, several critical gaps remain unaddressed. First, most studies focus on either optimizing renewable energy resources or managing DR programs independently without fully examining their combined effects within an integrated framework. Additionally, few studies consider the simultaneous interaction of DR programs, DGs, and ESSs to achieve optimal energy management and voltage stability under uncertainty. Finally, there is a clear need for more advanced models that integrate energy management, DR programs, and renewable resources to minimize operational costs while enhancing overall system performance. Finally, there is a clear need for more advanced models that integrate energy management, DR programs, and renewable resources to minimize operational costs while enhancing overall system performance.

This study aims to bridge these gaps by introducing a novel multi-objective optimization approach that simultaneously incorporates DR management, DG integration, and uncertainty handling. This framework is expected to significantly contribute to optimal energy management by reducing operational costs, improving voltage stability, and enhancing overall MG performance. Table 1 presents a structured comparison of various research studies on energy management and the optimization of distributed energy resources (DERs).

**Table 1:** Classification of specialized literature in the proposed research area.

Factors		References								This Paper
		[24]	[25]	[26]	[21]	[22]	[27]	[28]	[23]	
**Type of DERs**	**Renewable energy source**	✔	✔	✔	✔	✔	✔	✔	✔	✔
	**Micro Turbine**	✖	✖	✔	✔	✔	✖	✖	✖	✔
	**Diesel Generator**	✖	✖	✖	✔	✔	✖	✖	✔	✔
	**Energy storage system**	✖	✔	✔	✔	✔	✖	✔	✔	✔
**Type of End-user**	**Residential end user**	✔	Not Mentioned	Not Mentioned	✔	Not Mentioned	Not Mentioned	✔	Not Mentioned	✔
	**commercial end user**	✔	Not Mentioned	Not Mentioned	Not Mentioned	Not Mentioned	Not Mentioned	✔	Not Mentioned	✔
	**Industrial end user**	✔	Not Mentioned	Not Mentioned	✖	Not Mentioned	Not Mentioned	✖	Not Mentioned	✔
**Sensitivity Analysis**		✖	✖	✖	✖	✖	✖	✖	✖	✔
**Grid connect**		✖	✔	✔	✔	✔	✔	✖	✖	
**Power Losses**		✖	✖	✖	✖	✖	✖	✖	✖	✔
**Power Flow**		✖	✔	✖	✔	✖	✔	✔	✔	✔
**Voltage Deviation**		✖	✖	✖	✖	✖	✔	✖	✖	✔
**Demand Response**		✔	✔	✔	✔	✔		✔	✔	✔
**Cost Minimization**		✔	✔	✔	✔	✔	✔	✔	✔	✔
**Uncertainty**		✔	✖	✔	✔	✔	✔	✔	✔	✔

### 1.2. Contribution

Recent studies have often overlooked the uncertainties associated with wind and solar generation, microturbines, and the role of DR in power systems. To address this gap, we propose a stochastic optimization framework that integrates advanced algorithms to enhance the performance and resilience of MGs. The key contributions of this study are as follows:

- Optimization methods are introduced in order to enhance system flexibility by considering various scenarios for distributed generation sources. This ensures CM and improves MG performance.
- EVs are used as ESS, and their operation is optimized alongside the dynamic management of ESS and RES.
- DR considerations are incorporated into MG systems, leading to more effective system optimization. Additionally, technical indicators such as VD and the prevention of short-term level fluctuations, are accounted for to maintain system stability and protect against disturbances while ensuring economic optimization.
- The system’s responsiveness to the changes in the input information is emphasizes, providing insight into uncertainties associated with natural conditions and key technical network parameters.
- A DR program is proposed, operating through a Time-of-Use Pricing and incentive-based and punitive scheme, with a pricing mechanism informed by consumer awareness.
- A load accountability initiative is developed as a comprehensive, multi-tiered program featuring diverse incentives and penalty mechanisms, applied based on consumers’ usage relative to the defined standard consumption threshold.

This paper is organized as follows: Section 2 presents the proposed conceptual framework, Section 3 describes the mathematical formulation, Section 4 explains the simulation process and discusses the obtained results, and finally, Section 5 summarizes the key findings and conclusions.

## 2. Proposed framework

**Fig 1:**
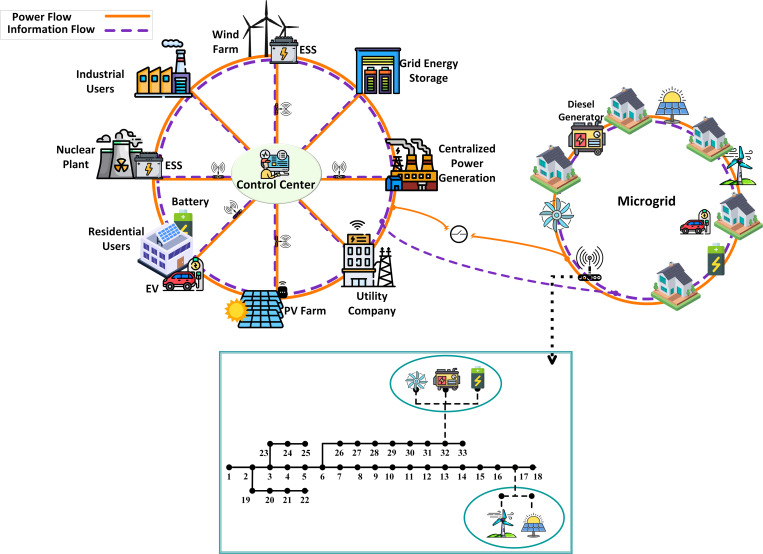
Graphical conceptual model for proposed optimization.

Fig 1 shows the proposed MG structure, which is interconnected with the upstream grid and supported bidirectional power flow. The energy demand of MG can be supplied either by the upstream grid or through locally DERs. To address uncertainties, a scenario-based stochastic optimization approach is implemented. Any surplus can be stored in a shared storage system or injected back into the upstream grid to enhance operational efficiency. The proposed framework is designed to accommodate the energy requirements of industrial, commercial, and residential consumers. To address uncertainties, a scenario-based stochastic optimization method is implemented. The studied system is a standard 33-bus network, incorporating a small-scale distributed energy system. PV and wind energy sources are integrated at bus 17, while energy storage systems, diesel generators (DIGs), and microturbines are installed at bus 32. The dataset includes distribution network parameters along with details of the distributed generation resources.

## 3. Mathematical problem formulation

This approach prioritizes secure and efficient EM scheduling while enhancing technical indices and system reliability. A multi-objective optimization algorithm developed to solve the formulate mathematical model, which will be detailed in the following sections. During the optimization process, several fundamental parameters are considered as inputs to the model, including load profiles (representing energy consumption), electricity prices, the network’s initial configuration, initial DG output, and initial heating, cooling, lighting, and V2G loads. The objective is to minimize costs while improving network efficiency, ensuring higher stability and better energy management. A hierarchical control system plays a crucial role in optimizing the operation of distribution networks and MGs. This system consists of three control levels:

- Primary control operates at the local level in real time, adjusting voltage and frequency.
- Secondary control ensures coordinated operation among MG nodes, improving overall system stability and performance.
- Tertiary control focuses on long-term tasks, including energy dispatch, resource allocation, and interaction with the main grid.

This multi-layered control structure allows the system to respond flexibly to changes in demand, generation, and external conditions, ensuring reliable and efficient operation. To enhance real-world applicability, uncertainties are introduced into the input data, simulating unpredictable variations in load profiles, renewable energy generation, electricity prices, and other key variables. The optimization algorithm operates across multiple scenarios to ensure robustness against sudden changes, thereby improving network reliability and stability. Additionally, key technical indices, such as the initial loss index, VD index, load ability limits (LAL), state of charge of ESS, and DG limits, are incorporated into the model. These indices provide a comprehensive overview of the network’s current state and serve as the critical inputs for the optimization algorithm to make optimal decisions regarding energy management, network stability, and overall efficiency. Finally, Algorithm 1 provide a systematic approach to optimizing DR in MGs under Uncertainty. To enhance accuracy, real-time data collected from DMUs (including voltage magnitudes and angles at each bus) are integrated into the model. These real-time inputs enable immediate system adjustments and facilitate the selection of the most effective DR strategy. Consequently, the system can dynamically apply optimal energy management techniques while maintaining network stability under varying conditions.

**Algorithm 1:** Hierarchical Control and Optimization Framework for Real-Time DR in Smart MGs Under Uncertainty

**Start**

**Load Profile ()** ***%Load Profile and Parameters***

 • Electricity Price = Get Electricity Price ()

 • Initial_ Reconfiguration = Get Initial Reconfiguration ()

 • Initial_ DG_ Generation = Get Initial DG ()

 • Initial_V2G_Loads = Get Initial V2G Loads ()

 • Initial_ Cooling_ Loads = Get Initial Cooling Loads ()

 • Initial_ Lighting_ Loads = Get Initial Lighting Loads ()

**Step 1: Calculating Required Parameters for Optimization**

 • Calculate Required Parameters ()

 if Electricity Price > Threshold:

  Adjust Load Profile ()

 else:

 • Maintain Current Profile ()

**Step 2: Hierarchical Control System**

 • Implement Hierarchical Control System ()

 • for each subsystem in MG: Control Subsystem (subsystem)

**Step 3: Applying Uncertainty to All Input Data**

 • Apply Uncertainty to Input Data ()

 • for each parameter in Input Data:

 • parameter = parameter * (1 + Random Uncertainty Factor ())

**Step 4: Preparing Data for Optimization Algorithm**

 • Prepare Optimization Data ()

 Required_ Data = **{**

 • Losses_ Index = Calculate_ Losses Index (),

 • Voltage_ Deviation_ Index = Calculate_ VD (),

 • ENS_ Index = Calculate ENS (),

 • Load_ Utility_ Limit_ Index = Calculate Load Utility Limits (),

 • SOC_ESS = Get State of Charge ESS (),

 • DG_ Limits = Get DG Limits ()

 **}**

 if any (value == NULL in Required _ Data):

  Throw Error (“*Missing Data for Optimization*”)

**Step 5: Optimize the Proposed Objective Function**

 • Optimize Objective Function ()

 • Initialize Optimization Algorithm ()

 • for each iteration in Optimization Algorithm:

 • Evaluate Objective Function ()

 • Update Parameters ()

 if Convergence Criteria Met ():

  break

**Step 6: Using Proposed Algorithm for Optimal DR**

 • Use Proposed Algorithm ()

 if Optimization Results Are Feasible ():

 • Apply Optimal DR ()

 else:

 • Log Error (“*Optimization Results Not Feasible*”)

**Step 7: Reapplying Hierarchical Control System**

 • Implement Hierarchical Control System ()

 • for each subsystem in MG: Update Control Subsystem (subsystem)

**Step 8: Reapplying Uncertainty to All Input Data**

 • Apply Uncertainty to Input _Data ()

 • for each parameter in Input _Data:

 • parameter = parameter * (1 + Random Uncertainty Factor ()

**Step 9: Re-preparing Data for Optimization Algorithm**

 • Prepare Optimization Data ()

 Required _Data = {

  Losses _Index = Calculate Losses Index (),

  Voltage _Deviation_ Index = Calculate VD (),

  ENS_ Index = Calculate ENS (),

  Load_ Utility_ Limit_ Index = Calculate Load Utility Limits (),

  SOC_ESS = Get State of Charge ESS (),

  DG_ Limits = Get DG Limits ()

 }

**Step 10: Setting the Optimal DR Using Proposed Algorithm**

Set Optimal DR ()

 Real_ Time Input Data = Get Real Time Data ()

 for each bus in Real Time Input Data:

  Voltage _Magnitude = Calculate Voltage Magnitude(bus)

  Voltage_ Angle = Calculate Voltage Angle(bus)

Stop

### 3.1. Wind turbine power generation

In this study WTs are connected to bus 17 and the power generation by a WT is a function of two parameters of wind speed and type of WT which is calculated as follows [29]:


$$P_{wt,t}=\left\{ \begin{array}{c} 0\;if\;v_{t}\leq \;v_{min}\;or\;v_{t}\leq \;v_{max}\;\\ \theta +\;\lambda \;\times \;V_{t}+V^{t}+\gamma \;\times \;{{(V}_{t})}^{2\;}\;if\;v_{min}\;{\leq v}_{t}\leq \;v_{ws}\\ P_{wt}\;if\;v_{ws}\;{\leq v}_{t}\leq \;v_{max} \end{array}\right.\;$$ (1)

where ($P_{wT,t}$) denotes the generated power, ($v_{\min}$) represents the cut-in wind speed (the lowest threshold (3 m/s) at the WT begins power generation), $v_{max}$ is the cut-out wind speed (the upper limit beyond which the WT cease operation to prevent damage to its blades (25 m/s)). And normal operating range $v_{ws}$ is nominal wind speed (15 m/s), at which the turbine reaches its rated power. Additionally, θ, λ and γ are coefficients specific to the wind turbine. The power generation of a WT ($P_{wT,t}$) denoted on the wind speed ($v_{t}$) and operates under distinct conditions according to specific wind speed thresholds. The relationship is defined in three main regions:

- **Cut-off Condition:** When the wind speed ($v_{t}$) is either below the minimum threshold ($v_{min}$) or exceeds the maximum limit ($\begin{array}{c} v_{max} \end{array}$), the power output is zero. where, ($v_{min}$) refers to the cut-in speed, and ($v_{max}$) denotes the cut-out speed.
- **Normal Operating Range:** In the range $v_{\min}\;{\leq v}_{t}\leq \;v_{ws}$, the turbine’s power output follows a polynomial function based on empirical coefficients:

$$P_{wt,t}=\theta +\;\lambda \;\times \;V_{t}+V^{t}+\gamma \;\times \;{{(V}_{t})}^{2\;}\;$$ (1.1)

- **Constant Power Range:** When wind speed is between $v_{ws}\;$and $\;v_{max}$, the turbine operates at a constant power output, denoted $\;P_{wt}$, which is the rated power of the turbine.

### 3.2. Photovoltaic power generation modeling

In this study PV panels are connected to bus 17. The power generation is modeled as follows:

The power output of PV panels, $P_{PV}(t)$, depends on environmental conditions and panel specifications. Key parameters include the number of panels$\;N_{sp}$, cell voltage ${\;V}_{sp}$ and ${\;I}_{sp}$ current, and the fill factor (FF), which represents the conversion efficiency of the PV cells. The power generation is expressed as:


$$P_{PV}(t)=\;N_{sp}{\;\times \;V}_{sp}{\;\times \;I}_{sp}{\;\times \;FF\;}_{.}\;\forall \in t$$ (2)

The FF is Calculated as the ratio of the maximum power point values to the open-circuit voltage and short-circuit current:


$$\;FF=\frac{V_{Ml}}{V_{OC}}\;\times \;\frac{I_{Ml}}{I_{SC}}$$ (3)

where, $V_{Ml}\;$ and $I_{Ml}$ are the voltage and current at the maximum power point, while $V_{OC}\;$ and $I_{SC}$ represent the open-circuit and voltage and short-circuit current, respectively. The voltage and current can be further adjusted for temperature effects as follows:


$$V^{t}{=\;V}_{OC}-\rho_{T}\left(\;AT+{SR}^{t}\left(\;\frac{N_{OT}-20}{0.8}\;\right)\right)$$ (3.1)

$$\;I^{t}\;={SR}^{t}[\;I_{SC}+\;\rho_{T}\left(\;T_{C}^{t}\;-25\right)\backslash ]$$ (3.2)

In these equations, $\rho_{T}$ is the temperature coefficient, and $\;T_{C}$ is the cell temperature. These adjustments improve the model’s accuracy by incorporating the effects of solar radiation (SR) and temperatures on PV panel performance. For further details, refer to [21].

### 3.3. Enhanced uncertainty modeling for optimal microgrid operation

This paper’s approach to a decentralized DR model for an optimal multi-objective energy management is based on managing the uncertainties of the renewable energy generation, consumer load, and other environmental factors in microgrid systems [30] These uncertainties are due to the stochastic characteristics of wind turbine power ($P_{WT}$), Photovoltaic power ($P_{PV}$), Microturbine power, Diesel generator power, Energy Storage Systems (ESS), and time varying loads such as heating, cooling, lighting and vehicle to grid (V2G) services. The abundant variability that accompanies these factors renders the problem of energy management extremely challenging, in terms of both reliability and costs. This section addresses these obstacles by introducing a novel combined uncertainty model for robust adaptive optimization strategies, anchored by a newly derived combined uncertainty formula (Equation 4), which is aimed at improving optimization within parameter boundaries that change with time. The uncertainty model we propose takes the form of:


$$\sigma_{ij}=\sqrt{\left(\sum_{i=1}^{n}\sum_{j=1}^{n}w_{i}w_{j}\left(\frac{\partial f}{\partial x_{i}}\frac{\partial f}{\partial x_{i}}.\;\sigma_{x_{i}}.\sigma_{x_{j}}.\rho_{ij}\right)+\sum_{i=1}^{n}\sum_{j=1}^{n}\left(\frac{\partial^{2}f}{\partial x_{i}\partial x_{i}}.\sigma_{x_{i}}.\sigma_{x_{j}}\right)\right)\;}\;$$ (4)

This equation combines first-order sensitivities, second-order effects, and variable weights to model the relationship and dependence of the input variables which becomes the basis for stochastic optimization in the presented microgrid framework.

- **Detailed Uncertainty Modeling**

In the microgrid context, uncertainty stems from the variability in the distributed generation sources such as wind turbines ($P_{WT}$) at bus 17, photovoltaic panels ${(P}_{PV}$) at bus 17, microturbines, diesel generators at bus 32, and other consumer loads like (e.g.,$\;P_{H_{C}},P_{C_{C}},\;P_{{v2g}_{C}},P_{l}$) and some external elements like wind speed ($V_{t}$), solar radiation (SR), and ambient temperature (AT). These wi uncertainties are addressed in this equation through weighted contributions *wi* that grants a modeling power for every single variable, correlation coefficients ($P_{ij}$) for interdependences (e.g., between $P_{WT}$ and $P_{PV}$), along with second order coefficients to account non-linear influences. This guarantees a complete portrayal of uncertainty to meet the aim of the study of making grid operation more stable, cost effective, and environmentally friendly under different conditions.

#### Specification of probability distributions.

The stochastic input variable actions are represented and calculated using their historical data and structural features (as seen in Table 8):

**Table 2:** Cost-benefit summary for the 33-bus system (10-year horizon).

Category	Cost/Benefit ($)
Capital Costs	1,707,840
O&M Costs (10 years)	708,710
Total Costs	2,416,550
Grid Purchase Savings	1,500,000
Emission Reduction Benefits	650,000
Reliability Benefits	1,830
Total Benefits	2,151,830
Net Benefit (Undiscounted)	−264,720
NPV (5% discount rate)	8,842

**Table 3:** Comparison of optimization methods (IEEE 118-bus network).

Optimization Method	Indexes						
	Number of Iteratind	CPU Times (s)	Totoal Cost ($)	Total Losses (kW)	Total DR (kW)	Voltage deviation (pu)	(EENS) (kWh)
**(PM)**	**20**	**3412**	**1233.627**	**3577.68**	**2495.638**	**0.1517**	**122.32**
**(PSO)**	30	3,452	3989.291	7086.56	2320.450	0.7599	241.08
**(MODE)**	56	3512	3304.747	6432.94	2385.720	0.7456	221.08
**(MOGA)**	115	4573	2840.776	9391.41	2290.300	0.7169	235.31
**(MOBE-PSO)**	40	5056	2080.213	5985.74	2435.880	0.7155	228.26

**Table 4:** Comparison of key features of optimization algorithms.

Feature	Proposed Method (PM)	PSO	MODE	MOGA	MOBE-PSO
**Search Strategy**	Hybrid approach combining scenario-based stochastic optimization and adaptive tuning	Swarm-based search with fixed cognitive and social parameters	Differential evolution with adaptive mutation and crossover	Population-based genetic search with crossover and mutation	Biogeography-based optimization integrated with PSO
**Convergence Speed**	Fast (20 iterations)	Moderate (30 iterations	Moderate (56 iterations)	Slower (higher iterations due to population-based search)	Moderate (40 iterations)
**Computational Complexity**	reduced by real-time feedback and adaptive tuning	depends on swarm size and fixed parameters	depends on population size and mutation strategy	High, due to population-based operations and crossover/mutation	hybrid approach increases complexity slightly
**Key Advantage**	Balances exploration and exploitation, adapts to real-time system changes	Simple implementation, effective for small to medium systems	Robust for multi-objective problems with adaptive search	Robust for multi-objective problems, but computationally intensive	Combines PSO’s simplicity with biogeography-based global search
**Key Limitation**	Requires real-time data integration for optimal performance	Sensitive to parameter settings, less adaptive to dynamic changes	Sensitive to initial population and mutation rates	High computational cost, less efficient for large-scale systems	Increased complexity due to hybrid structure, requires tuning

**Table 5:** Amounts of consumption and generation in every hour of the day for 33-Bus System.

Hours	PV (kW)	Wind Turbine (kW)	Diesel (kW)	Micro Turbine (kW)	Battery (kW)	New Demand Load (kW)	Demand Response (kW)	Utility (kW)
1	0	51.87458	4.15E-07	48.12471	−49.9983	50.00196	108.7357	3.91E-07
2	0	53.30772	8.21E-07	46.69134	−49.9981	50.00193	118.9613	4.2E-08
3	0	52.47069	5.68E-12	47.5292	−49.9989	50.00198	76.67971	8.97E-07
4	0	48.85951	1.83E-07	4.69E-07	1.140492	50.001	99.65408	1.37E-07
5	0	36.48653	3.39E-11	1.09E-07	13.51346	50.00099	96.43901	6.83E-11
6	87.7485	11.29212	0.000254	11.80949	17.06691	127.9203	0.000473	0.002318
7	99.92032	13.76218	0.001167	0.149809	26.52077	140.3573	0.026501	0.002095
8	105.9379	5.71544	0.0012	0.012993	13.19579	124.8662	0.007069	0.001916
9	80.05432	28.2437	8.38E-05	0.003944	42.47671	150.7822	0.001769	0.002414
10	108.4713	8.889296	0.001406	1.42522	20.73583	139.5414	0.003595	0.017388
11	92.90257	2.64E-06	1.86E-07	3.81E-08	−0.00096	92.90261	2.61E-07	1.28E-07
12	112.3597	10.56613	3.02E-05	0.000308	11.9252	134.853	0.000264	0.001472
13	115.4196	0	1.15E-14	8.76E-14	−0.001	115.4196	6.64E-10	1.89E-15
14	103.0216	1.28E-07	1.93E-08	4.41E-08	−0.00098	103.0216	5.77E-08	3.54E-08
15	122.3939	0	2.27E-12	9.39E-12	−0.001	122.3939	3.51E-13	1.92E-11
16	100.735	1.14E-14	1.53E-12	4.36E-12	−0.001	100.735	0	0
17	113.3521	0.000331	3.1E-06	3.08E-06	1.000749	114.3542	1.77E-05	6.39E-06
18	125.3197	1.97E-06	1.41E-06	5.94E-06	−0.00051	125.3202	6.59E-07	1.95E-06
19	109.8751	4.994486	0.000997	7.399615	9.689637	131.9636	0.00781	0.003087
20	111.3876	8.914386	0.00338	0.001046	14.68019	134.9891	0.004718	0.001443
21	0	71.50792	1.51E-14	28.49166	−49.999	50.00142	70.92756	7.73E-06
22	0	64.19298	6.34E-10	35.80607	−49.9981	50.00195	60.01886	1.84E-08
23	0	16.44029	20.10002	25.10002	15.10002	106.8404	37.04039	30.10002
24	0	19.85414	21.46238	26.46238	16.46238	115.7037	48.62842	31.46238

**Table 6:** Hourly consumption and generation in the 69-bus system.

Hours	PV (kW)	WT (kW)	Diesel (kW)	MT (kW)	Battery (kW)	New Load (kW)	DR (kW)	Utility (kW)
1	0	53.0675	4.23E-07	49.08720	−54.9980	45.9977	111.0014	3.99E-07
2	0	54.5352	8.37E-07	47.62516	−54.9980	44.9979	121.3405	4.28E-08
3	0	53.6772	5.79E-12	48.47978	−54.9980	44.9980	78.21330	9.15E-07
4	0	49.9837	1.87E-07	4.78E-07	−54.9980	24.8351	101.6472	1.40E-07
5	0	37.3079	3.46E-11	1.11E-07	−54.9980	12.2143	98.36779	6.97E-11
6	89.7810	11.5518	2.59E-04	12.04568	54.9980	168.0614	4.83E-04	2.36E-03
7	102.1635	14.0755	1.19E-03	0.152805	54.9980	171.3890	0.027031	2.14E-03
8	108.3210	5.8467	1.22E-03	0.013253	54.9980	169.1780	0.007210	1.95E-03
9	81.8738	28.8945	8.55E-05	0.004023	54.9980	165.7685	0.001805	2.46E-03
10	110.9142	9.0904	1.43E-03	1.453724	54.9980	176.4558	0.003667	0.017736
11	94.9999	2.70E-06	1.90E-07	3.89E-08	0.9473	95.9472	2.66E-07	1.31E-07
12	114.8901	10.8099	3.08E-05	0.000314	0.9473	126.6477	0.000269	1.50E-03
13	118.0356	0	1.17E-14	8.94E-14	0.9473	118.9829	6.77E-10	1.93E-15
14	105.3472	1.31E-07	1.97E-08	4.50E-08	0.9473	106.2945	5.89E-08	3.61E-08
15	125.2090	0	2.32E-12	9.58E-12	0.9473	126.1563	3.58E-13	1.96E-11
16	103.0173	1.16E-14	1.56E-12	4.45E-12	0.9473	103.9646	0	0
17	115.9356	0.000339	3.16E-06	3.14E-06	0.9473	116.8832	1.81E-05	6.52E-06
18	128.1591	2.02E-06	1.44E-06	6.06E-06	0.9473	129.1064	6.72E-07	1.99E-06
19	112.3689	5.1076	0.001019	7.56661	54.9980	180.0422	0.007966	0.003149
20	113.9157	9.1149	0.003456	0.001069	54.9980	178.0332	0.004812	0.001472
21	0	73.1555	1.54E-14	29.14149	−54.9980	45.9976	72.37311	7.89E-06
22	0	65.6732	6.47E-10	36.63119	−54.9980	45.9970	61.21924	1.88E-08
23	0	16.8166	20.56292	25.67802	15.40202	108.9772	37.78020	30.70202
24	0	20.3050	21.95653	27.07762	16.79163	118.0178	49.61199	32.09162

**Table 7:** Hourly consumption and production in the 118-bus system.

Hours	PV (kW)	WT (kW)	Diesel (kW)	MT (kW)	Battery (kW)	New Load (kW)	DR (kW)	Utility (kW)
1	0	180.5233	1.44E-06	167.4737	−69.9980	149.9960	378.6145	1.36E-06
2	0	185.5115	2.86E-06	162.6125	−69.9980	149.9960	414.1995	1.46E-07
3	0	182.5978	1.98E-11	165.4752	−69.9980	149.9960	266.8915	3.12E-06
4	0	170.0506	6.37E-07	1.63E-06	−69.9980	99.9960	346.8488	4.77E-07
5	0	126.9736	1.18E-10	3.79E-07	−69.9980	56.9716	335.6950	2.38E-10
6	305.3645	39.30378	8.84E-04	41.1021	69.9980	455.7692	1.65E-03	8.07E-03
7	347.7173	47.88966	4.06E-03	0.521533	69.9980	466.1305	0.092246	7.30E-03
8	368.6640	19.89264	4.16E-03	0.045225	69.9980	458.6038	0.024597	6.66E-03
9	278.5891	98.29491	2.92E-04	0.013728	69.9980	446.8959	0.006159	8.40E-03
10	377.4800	30.93400	4.88E-03	4.961230	69.9980	483.3780	0.012514	0.060528
11	323.4067	9.19E-06	6.48E-07	1.33E-07	−11.4733	311.9335	9.08E-07	4.47E-07
12	391.0710	36.77176	1.05E-04	0.001072	−11.4733	416.3706	9.18E-04	5.12E-03
13	401.6861	0	3.99E-14	3.05E-13	−11.4733	390.2128	2.31E-09	6.58E-15
14	358.4803	4.47E-07	6.72E-08	1.54E-07	−11.4733	347.0070	2.01E-07	1.23E-07
15	426.4365	0	7.92E-12	3.27E-11	−11.4733	414.9632	1.22E-12	6.69E-11
16	350.5587	3.89E-14	5.32E-12	1.52E-11	−11.4733	339.0854	0	0
17	394.4658	0.001152	1.08E-05	1.07E-05	−11.4733	382.9937	6.17E-05	2.22E-05
18	436.1495	6.86E-06	4.92E-06	2.07E-05	−11.4733	424.6762	2.29E-06	6.79E-06
19	382.3665	17.37877	0.003469	25.75251	69.9980	495.4993	0.027189	0.010744
20	387.6573	31.01647	0.011768	0.003641	69.9980	488.6872	0.016421	5.02E-03
21	0	248.8750	5.26E-14	99.16985	−69.9980	149.9960	246.9757	2.69E-05
22	0	223.4198	2.21E-09	124.6611	−69.9980	149.9960	208.9660	6.42E-08
23	0	57.21114	69.97207	87.35257	49.99900	371.9290	128.9450	104.7721
24	0	69.09483	74.68037	92.08255	49.99900	402.7607	169.2808	109.5771

**Table 8:** Total power generation and associated costs for 33-bus, 69-bus, and 118-bus networks from simulation results.

Components	Parameter	33-bus	69-bus	118-bus
PV	$P_{Total}^{PV}(kW)$	1588.899	1620.677	5529.368
	${Cost}_{Total}^{PV}(\$)$	25.36434	25.87163	88.2679
WT	$P_{Total}^{WT}(kW)$	507.3724	517.520	1765.656
	${Cost}_{Total}^{WT}(\$)$	25.72741	26.24196	89.53139
DIG	$P_{Total}^{DG}(kW)$	41.57092	42.40234	144.6668
	${Cost}_{Total}^{DG}(\$)$	103.8949	105.9728	361.5543
MT	$P_{Total}^{MT}(kW)$	279.0078	284.587	970.9471
	${Cost}_{Total}^{MT}(\$)$	147.8262	150.7827	514.4352
ESS	$P_{Total}^{ESS}(kW)$	-46.4898	−47.4196	−161.7845
	${Cost}_{Total}^{ESS}(\$)$	81.49388	83.12376	283.5987
DR	$P_{Total}^{DR}(kW)$	717.1373	731.480	2495.638
	${Cost}_{Total}^{DR}(\$)$	58.80526	59.98137	204.6423
Utility	$P_{Total}^{Utility}(kW)$	61.59455	62.82644	214.349
	${Cost}_{Total}^{Utility}(\$)$	28.98916	29.569	100.8823
Total	${Total}_{P}(kW)$	2416.85	2622.49	12941.85
	${Total}_{Cost}(\$)$	354.4906	361.5804	1233.627

**Fig 2:**
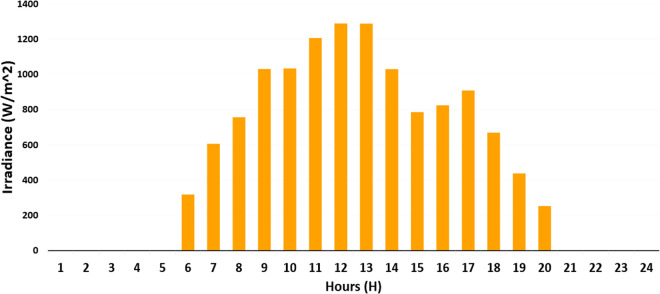
Solar irradiance (W/m^2^) across different hours of the day.

**Fig 3:**
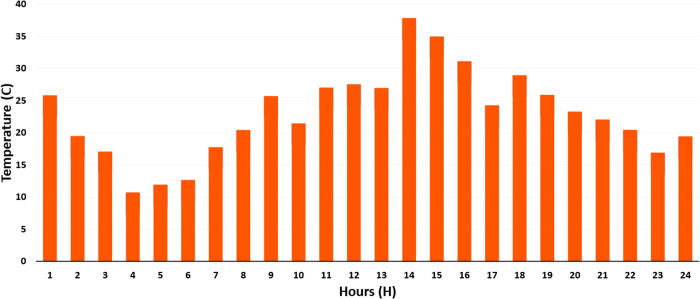
Hourly temperature (°C) data throughout the day.

**Fig 4:**
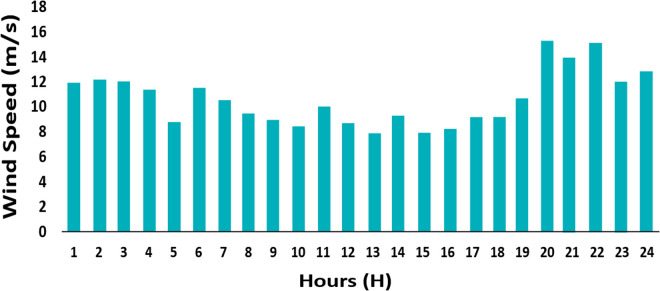
Wind speed (m/s) at different hours of the day.

- $P_{WT}:$ It is approximated as a Weibull distribution with k = 2.0 and λ = 8.0 m/s. It models variability in wind speed as per equation (1) and Fig 4.
- $P_{pv}$: It uses a Beta distribution with parameters of α = 2.5 and β = 5.0. Its scale is based on the effects of solar radiation and temperature as per equations (2) and (3) and Figs 2 and 3.
- $P_{load}:$ Initially, it is assumed to follow normal distribution with mean value of μ = 100 kW and standard deviation of σ = 20 kW. It captures various heating, cooling, V2G, and lighting loads fluctuation as shown in Figs 6 and 7. These distributions are customized to the 33-bus system’s operational data so that they match the objective of the study which is centered around real-time variability of load and generation data.

#### Scenario generation process.

Scenarios for stochastic optimization have been formed through an improved Monte Carlo simulation, enhanced through Latin Hypercube Sampling (LHS) which provides unbiased and efficient sampling of the probability space. The process is as follows:

- **Step 1**: For each of the 1000 random samples of each variable ($P_{WT},\;P_{PV},\;P_{load}$), out of which, 1000 samples are generated based on the probability distributions from Table 8.
- Step 2: Divide the entire distribution range into 100 equiprobable intervals where one sample is randomly taken from each interval to minimize sampling bias using LHS.
- Step 3: Constructed a correlation matrix using historical data and Pearson correlation coefficients ($\rho_{ij}$), for example, $\rho_{P_{WT},\;P_{PV}}$=0.3 (indicating weather correlations), $\rho_{P_{PV},\;P_{Load}}$=0.1 and $\rho_{P_{WT},\;P_{load}}$=0.05 based on the interdependencies presented in Figs 2–4.
- Step 4: Adjust samples to create a set of correlated samples by applying Cholesky decomposition of the correlation matrix.
- Step 5: Use equation (4) to compute and cluster the results into 10 representative scenarios with k-means clustering about each scenario with the assigned probability of 0.1 which depicts their occurrence in the 24-hour period. The methodology underlines a scenario-based stochastic optimization that improves energy management with variable inputs, as elaborated in Algorithm 2.

#### Update of selected parameters.

The parameters set in the uncertainty model and scenario generation are clearly stated in the following form:

- $w_{i}$: Weighting factors which indicate the importance of each variable, set as $W_{p_{wt}}$=0.3 $W_{p_{PV}}$=0.3, and $W_{p_{load}}$= 0.4, considering their impact on total system cost and load volatility (for example, heating loads at 59% in Fig 7).
- $\sigma_{x_{i}}:\;$Here the standard deviations relate to Table 8 and Fig 6. For Wind Power, $\sigma_{P_{WT}}$=15kW (pertaining to wind variability); for PV Panels, ${(\sigma }_{P_{PV}}$) = 10kW (on solar output variations); Load, $\sigma_{PLoad}$= 20kW (arising from uncertainty in load data).
- $\rho_{ij}$: The correlation coefficients have been defined as follows, $\rho_{P_{WT},\;P_{PV}}$= 0.3, $\rho_{P_{PV},\;P_{Load}}$=0.1 $\rho_{P_{wt},\;P_{Load}}$=0.05. These are based on the correlations of the environment and the demands.
- $\frac{\partial f}{\partial x_{i}}and\frac{\partial f}{\partial x_{i}}$: Calculated numerically based on the objective function $f=aP_{wt}^{2}+{bP}_{PV}+{cP}_{load}+{dP}_{DG}+{eP}_{MT}+gP_{ESS}$, $a=0.01\$/{kW}^{2}=2$,  b=0.02$/kW, c=0.03$/kW, d=0.05$/kW,  e=0.04$/kW, and g=0.015$/kW are cost coefficients derived from equations (20)–(26), reflecting the economic impact of distributed generation and storage.

This modeling framework is underpinned by empirical data and existing stochastic modeling captures the uncertainty and scenario generation in transparent detail. Aligned with the study goal of minimizing production and purchasing costs, whilst increasing voltage stability, decreasing power losses, and the reduction of environmental impacts.

### 3.4. Expected Energy not Served (EENS) criteria

As it pertains to planning and operational studies of electric power distribution systems, including microgrids, one of the key issues is assessing reliability of the network. From the many parameters that describe network reliability, the EENS index is commonly seen as the best and most useful for measuring reliability at different levels of energy supply and demand, and transmission automation. Its calculation is based on estimating the probabilities of forced outages for each of the system’s components which include generating units and power distribution lines. The work done in this thesis project deals with estimating the amount of load that is not supplied due to forced outages of the generating units and the distribution lines, considering the influence of a fictitious power system operator at each bus. To represent continuous events and their probabilities, a non-stochastic approach has been adopted. Then, the index is determined from the formula given in [31]


$$F_{i}=EENS=EENS_{G}+EENS_{L}$$ (5)

$$EENS_{G}=\sum_{i=1}^{NB}P_{i,d}\delta_{i}.$$ (5.1)

$$EENS_{L}=\sum_{i=1}^{NB}P_{j,d}\lambda_{j}$$ (5.2)

The aggregate EENS reflects the system’s reliability index (kWh) and is computed by adding together $EENS_{G}$, energy not supplied because of generation unit failures (kWh), and $EENS_{L}$, the energy not supplied because of distribution line faults (kWh). In this context, NB represents the number of buses in the microgrid (33 buses in the IEEE 33-bus system). The term $P_{i,d}$ is the probability of failure of the generation unit at bus i as obtained from uncertainty models (Section 3.3), while $\delta_{i}\;$is the demand deficit at bus i (kWh) that is defined as the gap between demand and the supply of energy during a failure. Likewise, $P_{j,d}\;$is the probability of failure of distribution line j and is acquired from line failure information, and $\lambda_{j}$is the load not supplied because of the failure of line *j* (kWh) which is calculated from the load that is connected to that line.

### 3.5. Multi-Objective optimization

This study achieves network optimization and cost reduction by considering the technical parameters stable operation. the optimization process minimizes significant changes in other parameters to maintain system consistency and avoid adverse effects. Key objectives include minimizing VD, implementing a multilevel load DR program based on shared consumption, and optimizing distributed generation sources over a 24-hour period. The total energy load consumption is calculated using Equation 6:


$$\;T_{l}=\sum_{1}^{i}\sum_{1}^{H}P_{H_{C}}+P_{C_{C}}+P_{{v2g}_{C}}+P_{l_{c}}$$ (6)

where $P_{H_{C}}$, $P_{{v2g}_{C}}$, $P_{l_{c}}$ represent heating, cooling, vehicle-to-grid, and other loads, respectively, all subject to uncertainty.

Active power losses are determined using equation 7:


$$P_{loss}=R.\frac{P^{2}+Q^{2}}{V^{2}}\;\times R$$ (7)

Reactive power losses are calculated with equation 8:


$$Q_{loss}=R.\frac{P^{2}+Q^{2}}{V^{2}}\;\times X$$ (8)

These formulations provide a robust framework for system managers to reduce power losses, improve system efficiency, and optimize overall performance in modern energy networks

### 3.6. Dual-dimensional demand response framework with time-of-use pricing and consumption-based incentive-penalty tiers

This study proposes an advanced DR framework that integrates two complementary operational dimensions temporal pricing via Time-of-Use (TOU) tariffs and consumption-based incentive-penalty tiers to optimize energy efficiency, enhance grid stability, and improve cost-effectiveness. The framework builds upon a six-tier structure, comprising three incentive tiers for reducing energy consumption, two penalty tiers for exceeding predefined limits, and a neutral tier applying the base electricity price. By incorporating a dynamic TOU pricing mechanism, this approach aligns consumer behavior with both grid demand patterns and individual usage efficiency, offering a robust and adaptive solution for modern energy management systems.

- **TOU Pricing Mechanism**

The first dimension of the framework employs a TOU pricing mechanism, which assigns variable electricity rates to distinct time periods within a 24-hour cycle, reflecting fluctuations in grid demand and generation costs. The base electricity price at any given hour t, denoted as $P_{g}(t)$ is determined by the following tariff structure:


$$P_{g}(t)=\;\left\{ \begin{array}{c} P_{off-peak,\;}if\;t\in Off-Peak\;Hours\;(e.g.,23:00-06:00)\\ P_{mid-peak,}\;if\;t\in Off-Peak\;Hours\;(e.g.,\;06:00-18:00)\;\\ P_{peak,}\;if\;t\in Off-Peak\;Hours\;(e.g.,\;18:00-23:00) \end{array}\right.\;$$ (9)

- $P_{off-peak\;}$: The base electricity price during off-peak hours, established at the lowest rate to encourage consumption when grid demand is minimal.
- $P_{mid-peak,}$: The base electricity price during mid-peak hours, set at a moderate rate to maintain load balance during periods of intermediate demand.
- $P_{peak,}$: The base electricity price during peak hours, fixed at the highest rate to discourage excessive usage and alleviate grid stress during high-demand periods.

This specific TOU tariff is implemented as the pricing primary layer, making $P_{g}(t)$a function of time of the day. The mechanism enhances system-wide efficiency while optimizing grid resource utilization, lowering operational costs, and changing loads to off-peak periods.

- **Consumption-Based Incentive and Penalty Tiers**

A six-tier structure is employed for the DR program that incentivizes to reduce energy consumption and punishes over the limit. For instance, three incentive tiers to reduce the reward and two penalty tiers, negative incentives, impose higher cost for excessive consumption. And there is a neutral tier that is a flat, base electricity price. Consumer costs depend on their hourly usage, which is influenced by some factors, such as the flat rate attributed to the TOU offered by the program, hourly usage, cap in usage, and incentive or punishment provided. Such an approach is aimed at achieving energy saving, sustainability of the grid and low price costs. The total cost incurred by a consumer is determined by their hourly consumption, as set forth in Equations (10–16). The intent of this multi-tiered system is to strive for increased energy efficiency without compromising the sustainability of the grid while ensuring costs remain low. Core factors are the base electricity price, $P_{g}$ the incurring expense of the consumer derived from their hourly spending, $E_{c}$, the limit in consumption, T, and incentive or punishment factor, $P_{r}$.

#### Level 1: Basic incentive.

At this level, a threshold for maximum load consumption is established. If the consumer’s usage falls within the specified range, they are eligible for an incentive, as outlined in Equation 8. the total cost is then calculated based on the duration the consumer-maintained consumption within this range. $if\;o\mathrm{.7}\;T<E_{c}\leq T$ the cost is calculated as:


$$\begin{array}{c} C_{1}=\;\sum_{t=1}^{t=24}P_{g}.E_{c,t}-\left(\left(P_{g}.T_{t}\right)+\left(P_{r,t}.E_{c,t}\right)-\left(P_{r,t}.T_{t}\right)-\left(P_{g}.E_{c,t}\right)\right) \end{array}$$ (10)

This formula calculates the total cost with this the incentive applied proportionally.

#### Level 2: Enhanced incentive.

At this level, the consumption range required to qualify for a higher incentive is narrower. If the consumer’s usage falls within this range, they will receive a greater incentive compared to the previous tier. $if\;o\mathrm{.5}\;T<E_{c}\leq 0.7T$ the cost is calculated as:


$$C_{2}=\;\sum_{t=1}^{t=24}P_{g}.E_{c,t}-\left(\left(P_{g}.T_{t}\right)+\left(1.3P_{r,t}.E_{c,t}\right)-\left(1.3P_{r,t}.T_{t}\right)-\left(P_{g}.E_{c,t}\right)\right)$$ (11)

The calculation follows the same structure as Equation 8, but with the incentive coefficient increased by 0.3 in Equation 9.

#### Level 3: Maximum incentive.

This tier offers the highest incentive. If the consumer’s consumption is below half of the designated threshold, they will receive the maximum incentive coefficient.

$if\;E_{c}\leq 0.5T$ the cost is calculated as:


$$C_{3}=\;\sum_{t=1}^{t=24}P_{g}.E_{c,t}-\left(\left(P_{g}.T_{t}\right)+\left(1.5P_{r,t}.E_{c,t}\right)-\left({1.5P}_{r,t}.T_{t}\right)-\left(P_{g}.E_{c,t}\right)\right)$$ (12)

This calculation follows the same structure as in the previous stages, with the incentive coefficient increased by a factor of 1.5. The costs for specific hours of the day are determined using Equation 10.

#### Level 4: Neutral consumption.

At this stage, if the consumer’s usage reaches up to 1.3 times the consumption threshold, they are required to pay according to the base price, with no incentive or penalty applied. $if\;1.3T>\;E_{c}>T$ the cost is calculated as:


$$C_{4}=\;\sum_{t=1}^{t=24}P_{g}.E_{c,t}$$ (13)

Penalty coefficients are applied consumer’s usage falls within this range.

#### Level 5: Basic penalty.

At this stage, if the consumer’s usage falls between the previous level and up to 1.5 times the consumption threshold, the cost is calculated using Equation 12. $if\;1.5T>\;E_{c}\geq 1.3T$ the cost is given by:


$$C_{5}=\sum_{t=1}^{t=24}P_{g}.E_{c,t}-\left(\left(P_{g}.T_{t}\right)+\left(P_{r,t}.E_{c,t}\right)-\left(P_{r,t}.T_{t}\right)-\left(P_{g}.E_{c,t}\right)\right)$$ (14)

The additional cost for excess consumption is calculated using Equation 12, where $P_{r}$ represents the penalty coefficient.

#### Level 6: Maximum penalty.

At this stage, if the consumer exceeds 1.5 times the consumption threshold, they will be required to pay the highest penalty. $if\;E_{c}\geq 1.5T$ the cost is given by:


$$C_{6}=\sum_{t=1}^{t=24}P_{g}.E_{c,t}-\left(\left(P_{g}.T_{t}\right)+\left(1.3P_{r,t}.E_{c,t}\right)-\left({1.3P}_{r,t}.T_{t}\right)-\left(P_{g}.E_{c,t}\right)\right)$$ (15)

The additional cost for excess consumption is calculated using Equation 13, with the penalty coefficient set at 1.3 times.

The total cost for the consumer is determined by calculated their consumption for each hour and applying the corresponding formula. The costs for all hours are then summed to obtain the total cost:


$$C_{DR}=\sum_{c=1}^{c=6}C_{c}$$ (16)

### 3.7. Voltage deviation optimization in the decentralized demand response framework

The effective control of VD is one of the primary goals within the scope of the model defined in this study, which was presented in Section 3.6 Dual-Dimensional Demand Responsive Framework. This framework captures the TOU pricing with a 6-level incentive and penalty system in order to minimize energy consumption while maximizing the stability of the grid. The deviation from normal voltage which is referred to as the difference between the bus voltage and its nominal value, is critical for overall system productivity and longevity. The first model for VD is stated as follows.


$$f^{2}=\sqrt{\sum_{1}^{B}(1-{V_{i})}^{2}\;}$$ (17)

In this case, B stands for the number of buses (IEEE 33-bus system) and $V_{i}\;$is the voltage magnitude (in p.u.) at bus i. This subsection seeks to further elaborate and improve this expression by providing the necessary detail on the formulation’s calculation and scope of definition. We consider the bus 1 of the IEEE 33-bus system where we set the nominal voltage serving as a computation basis of voltage variation at 12.66 kV (line to line) which equals to 1.0 p.u (per unit) in all buses for ideal scenario. In function f2, the constant “1” denotes $V_{nom}$=1p.u. Actual voltage at each bus is denoted by $V_{i}$. The purpose is to minimize the root mean squared deviation from the nominal voltage to achieve seamless network voltage profile. The VD limits have been set with compliance to IEEE Standard 1547 and current distribution system rules that require for voltage to be in a region of −5% to +5% of the nominal value (i.e., 0.95 p.u. to 1.05p.u) for reliable operation of the system. In this work the DR framework and DG optimization are designed to set limits on $V_{i}$, but unlike the previous approach the bounds are tighter at ±2% (0.98p.u to 1.02p.u) during the peak load time. The simulation results shown in Fig 10 of section 4 have been proven to stay within the described bounds, which confirms the stability of the system as the range between 0.01 and 0.02 p.u is much less than the acceptable limit of the system. To enhance precision, the VD objective is reformulated as:

**Fig 5:**
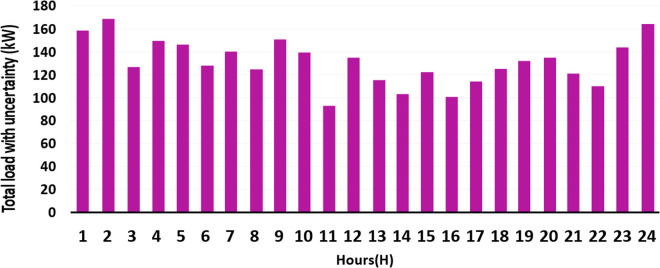
Total loads with uncertainty (kW) in each hour of the day.

**Fig 6:**
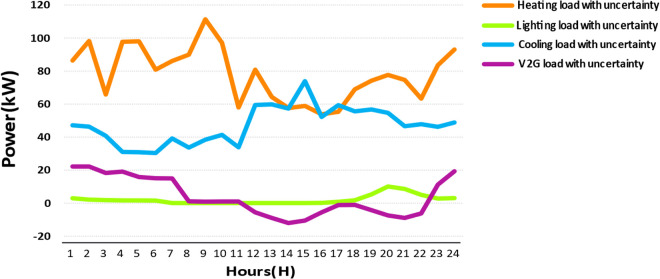
Input loads with uncertainty (kW) over a 24-hour period.

**Fig 7:**
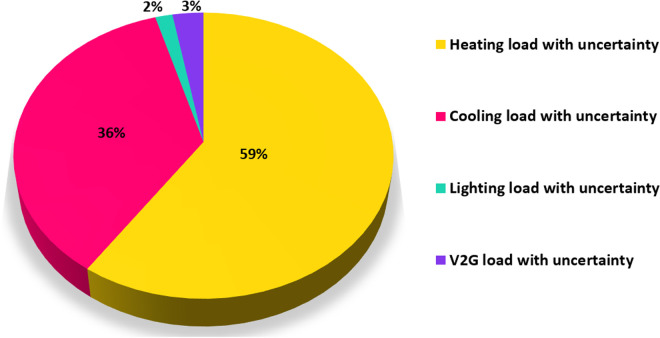
Distribution of input loads with uncertainty (percentage).

**Fig 8:**
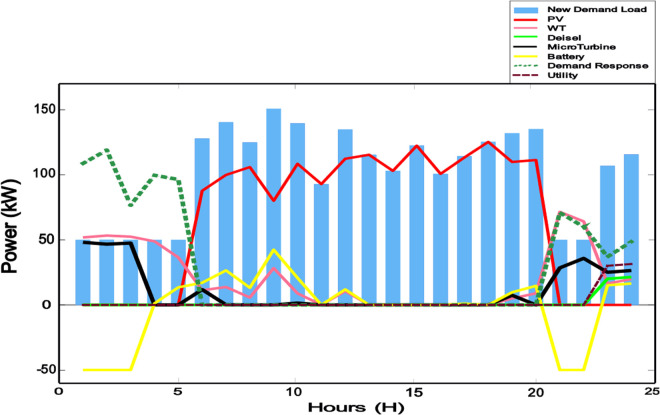
The amount of consumption and production in each hour of the day.

**Fig 9:**
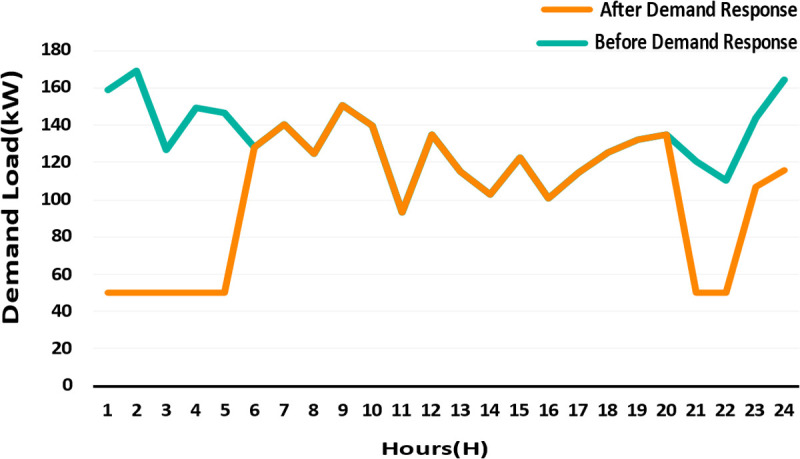
Demand load before and after implementing the demand response program.


$$f^{2}=\sqrt{\sum_{1}^{B}(V_{nom}-{V_{i})}^{2}\;},\;V_{nom}=1.0p.u\;$$ (18)

$${\;subject\;to\;the\;constraint:\;0.95p.u\leq V}_{i}\leq 1.05p.u$$

The value of $V_{i}$ is derived from hourly load flow analysis, incorporating the contributions of DG units (e.g., PV, WT, DIG, MT, ESS) and DR adjustments. The Particle Swarm Optimization algorithm, detailed in Algorithm 2, minimizes this function concurrently with cost-related objectives, ensuring voltage stability conforms to technical benchmarks. The DR framework reduces peak loads which helps in addressing the VD, leading to less voltage drop at remote buses such as buses 17 and 32 that have DG units. Furthermore, EVs aid in stabilizing voltage during peak demand by discharging stored energy back to the grid during V2G participation. This helps in reducing the voltage sags at important nodes. On the other hand, uncontrolled EV charging during peak hours contributes to enhancing the drop in voltage. Although unmanaged charging of EVs during peak hours leads to excessive voltage drop, actively managing the charge and discharge patterns of EVs is pivotal in ensuring that the voltage demand over the microgrid is met. For example, the starting demand at bus 32 is 200kW and the VD is 0.025 p.u. Based on historical DR data, when the demand is adjusted to 80 kW, the p.u VD improves to 0.015 p.u. This is an example of ‘cost optimization’ and ‘voltage stability’ working in unison as confirmed by the f2 objective.

Equation (19) represents the criteria, costs, and power outputs of each power generation source, including the ESS, while considering DR incentive over a 24-hour period. It calculates the overall cost for the consumer and offers insights for improving system performance and reducing coats. The equation is written as:


$$\begin{array}{c} T_{CN}=\left\{\left(\left(C_{PV\;}\times P_{PV}\right)+\left(C_{WT}\times P_{WT}\right)+\left(C_{DG}\times P_{Dg}\right)+\left(C_{MC}\times P_{MC}\right)+\left(\alpha \left(C_{B}\times P_{B}\right)\right)\right)\pm {(C}_{DR})\right\} \end{array}$$ (19)

Equation (20) calculates the sum of all network parameters, including those from equation (19), while considering VD, active and reactive powers, network costs. This objective function balances costs, power losses, VDs, and power variations in the system. The final equation is:


$$T_{C}=\;T_{CN}+P_{lossn}+Q_{lossn}+\;{\;V}_{D}\;$$ (20)

### 3.8. Cost modeling

Cost modeling for distributed generation sources, including PV, WT, BESS, DIG, MT, and the grid, are formulated in equations (21)–(27) [32–34].


$${cost}^{\zeta }=A\zeta +B\zeta \;\times P^{PW}(t)\;\forall \in T$$ (21)

$$A\zeta =\;\frac{{cost}_{capital}\times P_{capital\;}\times Gr}{T_{life}\times 365\times 24\times {CF}_{\zeta }}$$ (22)

$${cost}^{Bess}(t)=\;A_{Bess}+\left(B_{Bess}\;\times \;\left|P^{Bess}(t)\right|\right)\pm \left(\beta^{TOU}(t)\;\times \;P^{Bess}(t)\right)\forall \in T\;$$ (23)

$$A_{Bess}=\;\frac{{cost}_{Bess,c}\times P_{capital\;}\times Gr}{T_{life}\times 365\times 24\times {CF}_{\zeta }}\;;B_{Bess}={Cost}_{o\&m}^{\zeta }$$ (24)

$$\left\{ \begin{array}{c} {\;Cost}_{EMI}(t)=\;{(C}_{{CO}_{2}}+C_{{SO}_{2}}+C_{{NO}_{X}})\times P_{.}(t)\\ {Cost}_{O\&M}(t)\;=\;c\;+\;b\;\times P_{.}(t)+a\times P_{.}^{2}(t)\\ Cost=\;{Cost}_{O\&M}\;(t)\;+\;H_{.}\;\times {\;Cost}_{EMI}(t) \end{array}\right.\;{\begin{array}{c} \;\\ \;\forall \end{array}}_{.}\in \left\{DIG,MT,grid\right\}\;\forall_{t}\in T\;$$ (25)

$$\;H_{.}=\frac{{Cost}_{O\&M}^{.}(t)}{{Cost}_{EMI}^{.}(t)}|P^{\max}{\;\forall }_{.}\in \left\{DIG,MT,grid\right\}\;\forall_{t}\in T\;$$ (26)

$${Cost}_{Total}^{DIG}(t)=\;{Cost}_{O\&M}(t)+H\times {\;Cost}_{EMI}(t)+\frac{{Cost}_{cap}^{DIG}}{T_{life}\times 365\times 24}+\;C_{env}^{DIG}\;\forall_{t}\in T\;$$ (27)

The cost of distributed generation resources (PV, WT and BESS) consists of fixed and variable components. fixed costs depend on investment, device lifetime $T_{life}$, annual interest rate Gr, and capacity factor (CF). Variable costs include operational and maintenance costs. For controllable generators like MTs and DIGs, the total operating cost comprises emission and generation cost. Emission, primarily carbon dioxide (CO_2_), sulfur dioxide (SO_2_) and nitrogen oxides (NO_x_) are the main pollutants in the power grid. The fuel cost of DGs is modeled as a quadratic function. Production cost depend on capacity and real cost, with coefficients (a, b, c). the price penalty coefficient $H_{\xi \;}$ can transform these optimization problems into a two-objective function [34]. As defined in equation (21), based on the unit’s maximum output power. A diesel generator operating at 29.1 kW, which works for 5 hours a day during peak hours of use or when renewable energy is not available, has a fuel consumption rate of 10 liters an hour. This means that it emits 26.8 kg of CO_2_ per hour which calculates as fuel consumption multiplied by diesel gas emission per liter (10 liters × 2.68 kg/liter) [35]. The corresponding emission levels for NO_x_ and SO_2_ is at 0.000015 tons/kWh and 0.000001 tons/kWh, respectively [35].

The energy output for the given parameters is calculated by the formula 29.1 × 5 × 365, giving us 53,122.5 kWh per year. The yearly emissions for CO_2_, NO_x_, and SO_2_ stand at 48.91, 0.797, and 0.053 while being multiplied with their respective metric calculations (26.8 × 5 × 365 = 48,910 kg, 0.000015 × 53,122.5, and 0.000001 × 53,122.5). The cost of emissions for a market with the given prices of 2025 where $C_{{CO}_{2}}$=$50/ton, $C_{{CO}_{2}}$=$2,000/ton, $C_{{SO}_{2}}$= $1,500/ton [35], indicates that the savings made while operating at an efficiency per hour on CO_2_ emissions is at 0.00268 × 29.1 × 50 = $3.90, for NOx is 0.87, and 0.04 for SO_2_. Adding all prices results in $4.81 per hour, or $24.05 per day at ($4.81 × 5), giving us a final annual cost of $24.05 × 365 = $8,778, resulting in a total cost after 10 years at $8,778 × 10 = $87,780.This environmental cost of $87,780 is integrated into the operational cost model for DIG, ensuring the optimization framework accounts for both economic and environmental impacts, incentivizing reduced reliance on diesel generators in favor of renewable energy sources like PV and WT, which have negligible emission costs, thus aligning with sustainability goals and reducing the carbon footprint of the 33-bus system.


$$C_{env}^{DIG}(t)={(E}_{{CO}_{2}}\times P_{DIG}(t)+C_{{CO}_{2}})+{(C}_{{CO}_{2}}\times P_{DIG}(t)+C_{{NO}_{X}})+\left(E_{{SO}_{2}}\times P_{DIG}(t)+C_{{SO}_{2}}\right)$$ (28)

where ${(E}_{{CO}_{2}}$= 0.00268 tons/kWh,$\;E_{{NO}_{X}}$ = 0.000015 tons/kWh, $E_{{SO}_{2}}$= 0.000001 tons/kWh, $C_{{CO}_{2}}$= $50/ton, $C_{{NO}_{X}}$= $2000/ton, and $C_{{SO}_{2}}$= $1500/ton based on current emission markets.

### 3.9. Cost-benefit analysis of the proposed framework (Revised for economic feasibility in the 33-bus system)

In this part, the economic justification is covered for the decentralized DSR, along with the integration of multi-objective energy management strategies. An updated cost-benefit analysis was conducted for a 10-year period of operations for the 33-bus system and attempts to capture the revenue from the subsidized and less expensive technologies that are sure to strive towards positive Capital Expenditure (CAPEX) and Operational Expenditure (OPEX) values with a positive NPV, as shown in Table 2. The thesis presents a clear value structure including CAPEX value, O&M expenditure, and cost benefits such as savings and reduced environmental value along with improved dependability. Also, scalability for the 69-bus and 118-bus systems is discussed.

- ***Capital Costs***

Capital costs reflect the initial investments for deploying the proposed framework, optimized for cost reduction:

- *DG Units:* For photovoltaic disbursed generation panels of a maximum power output of 1430 kW, the total cost was $1,144,000 due to recent pricing drops of polycrystalline PV technology estimated at $0.80/W units. Wind turbine (WT) units with an output capacity of 431.2665 kW had a price of $560,646 estimated at $1.30/W Units. Microturbine units of 223.2062 kW had a cost of $100,443 giving a price of $450/kW and diesel generators of 29.1 kW were costed at $12,513 giving a price of $430/kW were obtained from [35].
- *ESS*: An 80 kWh lithium-ion battery energy storage system (BESS) with a 50 kW power rating costs $22,400 ($280/kWh), including installation, per updated estimates [36].
- *DR Infrastructure:* Smart metering and control systems for 33 buses are estimated at $24,750 ($750/bus), using cost-effective technologies [37].
- **Subsidies**: A $150,000 government subsidy for renewable energy installations is applied [38]. Total capital expenditure for the 33-bus system is $1,707,840 after subsidies.

- **Operational and Maintenance (O&M) Costs**

O&M costs are incurred annually over the 10-year period, optimized for efficiency:

- *DG Units:* PV and WT O&M costs are 1.5% of capital costs annually ($17,160 for PV, $8,410 for WT) [38,39]. MT costs are $0.015/kWh ($3,348/year for 223.2062 kW), and DG costs are $0.05/kWh ($1,455/year for 29.1 kW) [35].
- *ESS:* BESS maintenance is 2.5% of capital cost ($560/year) [36].
- *DR Program:* Maintenance of DR infrastructure is $150/bus/year, totaling $4,950/year for 33 buses [37]
- Total O&M costs over 10 years are $708,710, with an annualized cost of $70,871.

- **Economic and Environmental Benefits**

The benefits of the framework are enhanced through optimization:

- *Cost Savings from Reduced Grid Purchases*: Grid purchases are limited to 50 kW annually (hours 23–24), costing $23,500 at $0.047/kWh (2025 rate [40]). Without the framework, the 3715 kW load costs $174,605/year. Annual savings are $150,000, totaling $1,500,000 over 10 years.
- *Environmental Benefits:* Reducing DG usage to 20 kW cuts CO_2_ emissions by 1,300 tons/year, valued at $65,000/year ($50/ton, 2025 carbon price [35]), or $650,000 over 10 years.
- *Reliability Improvements: A* 45% EENS reduction (30.05 kWh to 16.53 kWh, Table 12) saves $1,830 over 10 years [41].

**Table 9:** Objective and technical parameters in the 33-bus system.

Hours	Objective Parameters				Technical Parameters			
	Load Before DR (kW)	Load After DR (kW)	VD Before (Pu)	VD After (Pu)	Active Losses Before (kW)	Active Losses After (kW)	Reactive Losses Before (kVar)	Reactive Losses After (kVar)
1	158.7377	50.00196	0.018001	0.019373	1.565995	0.426806	0.284018	1.346324
2	168.9632	50.00193	0.018956	0.019968	1.802476	0.473324	0.314975	1.550352
3	126.6817	50.00198	0.014419	0.015801	1.017012	0.273874	0.182245	0.874505
4	149.6551	50.001	0.017272	0.01792	1.336069	0.392948	0.261485	1.147396
5	146.44	127.9203	0.016203	0.016042	1.073118	0.345822	0.230124	0.918278
6	127.9207	127.9203	0.01374	0.011718	0.614695	0.248675	0.165476	0.521046
7	140.3838	140.3573	0.016639	0.012622	0.789626	0.364673	0.242669	0.670079
8	124.8733	124.8662	0.014081	0.013249	0.757731	0.261182	0.173799	0.647671
9	150.7839	150.7822	0.017438	0.011767	0.745794	0.400525	0.266528	0.627661
10	139.545	139.5414	0.016229	0.013392	0.837793	0.346921	0.230856	0.713103
11	92.90261	92.90261	0.010581	0.011289	0.527402	0.14748	0.098136	0.453111
12	134.8533	134.853	0.015282	0.014626	0.916225	0.307622	0.204703	0.783899
13	115.4196	115.4196	0.01354	0.013814	0.809649	0.241502	0.160703	0.695283
14	103.0216	103.0216	0.011403	0.012717	0.649684	0.171286	0.113977	0.558386
15	122.3939	122.3939	0.014282	0.014696	0.9101	0.268707	0.178806	0.781635
16	100.735	100.735	0.01124	0.01238	0.620794	0.166432	0.110747	0.533504
17	114.3542	114.3542	0.013291	0.0136	0.781032	0.232711	0.154853	0.670572
18	125.3202	125.3202	0.014135	0.015328	0.956829	0.26318	0.175128	0.822219
19	131.9714	131.9636	0.014991	0.013464	0.802164	0.296023	0.196984	0.684536
20	134.9938	134.9891	0.015236	0.014259	0.878718	0.305784	0.20348	0.750951
21	120.929	50.00142	0.013894	0.017993	1.266009	0.254297	0.169217	1.093476
22	110.0208	50.00195	0.012308	0.015707	0.95839	0.199553	0.132787	0.827064
23	143.8807	106.8404	0.016124	0.010396	0.519203	0.342469	0.227893	0.429413
24	164.3321	115.7037	0.017521	0.011331	0.717904	0.404367	0.269085	0.596834

**Table 10:** Objective and technical parameters in the 69-bus system.

Hours	Objective Parameters				Technical Parameters			
	Load Before DR (kW)	Load After DR (kW)	VD Before (Pu)	VD After (Pu)	Active Losses Before (kW)	Active Losses After (kW)	Reactive Losses Before (kVar)	Reactive Losses After (kVar)
1	156.987654	45.976543	0.0204	0.0163	3.218765	2.254321	2.147892	1.287654
2	166.345678	44.987654	0.0215	0.0172	3.376543	2.365432	2.254321	1.352678
3	123.198765	44.976543	0.0239	0.0191	3.543210	2.478654	2.365432	1.419876
4	126.478901	24.832109	0.0251	0.0201	3.465432	2.423876	2.309876	1.385432
5	110.576543	12.210987	0.0258	0.0206	3.487654	2.435210	2.321098	1.392654
6	168.065432	168.060987	0.0263	0.0210	5.554321	3.887654	3.698765	2.219876
7	171.412345	171.385432	0.0311	0.0156	6.187654	4.331098	4.127654	2.476543
8	169.182109	169.175432	0.0343	0.0172	4.843210	3.389876	3.231098	1.938654
9	165.767890	165.765432	0.0356	0.0178	5.012345	3.508654	3.341098	2.004321
10	176.456789	176.452345	0.0358	0.0179	5.112345	3.578654	3.409876	2.045432
11	95.943210	95.943210	0.0361	0.0181	5.154321	3.607890	3.432109	2.059265
12	126.645432	126.645432	0.0379	0.0190	5.465432	3.825876	3.641098	2.184654
13	118.987654	118.987654	0.0363	0.0182	5.154321	3.607890	3.432109	2.059265
14	106.290123	106.290123	0.0338	0.0169	4.723456	3.304321	3.147890	1.888734
15	126.152345	126.152345	0.0318	0.0159	4.376543	3.065432	2.921098	1.752654
16	103.960987	103.960987	0.0260	0.0208	3.698765	2.589012	2.465432	1.479265
17	116.879012	116.879012	0.0242	0.0194	5.354321	3.747890	3.567890	2.140734
18	129.102345	129.102345	0.0261	0.0209	5.554321	3.887654	3.698765	2.219876
19	180.047890	180.040123	0.0319	0.0160	6.443210	4.509876	4.294321	2.576543
20	178.035678	178.030987	0.0379	0.0190	7.498765	5.249012	4.998765	2.999265
21	118.367890	45.976543	0.0389	0.0195	5.632109	3.942476	3.752109	2.251265
22	107.212345	45.976543	0.0338	0.0169	4.754321	3.327890	3.167890	1.900734
23	146.754321	108.973456	0.0260	0.0208	3.712345	2.598654	2.473456	1.484074
24	167.625432	118.014567	0.0222	0.0178	3.532109	2.472476	2.352109	1.411265

**Table 11:** Objective and technical parameters in the 118-bus system.

Hours	Objective Parameters				Technical Parameters			
	Demand Load Before DR (kW)	Demand Load After DR (kW)	VD Before (Pu)	VD After (Pu)	Active Losses Before (kW)	Active Losses After (kW)	Reactive Losses Before D (kVar)	Reactive Losses After (kVar)
1	528.612345	149.997654	0.1442	0.1154	172.005432	120.403210	114.672345	68.803456
2	564.192345	149.995432	0.1501	0.1201	180.476543	126.332109	120.315432	72.189265
3	416.885432	149.997654	0.1612	0.1289	203.732109	142.612476	135.821098	81.492654
4	446.842109	99.994321	0.1658	0.1326	233.443210	163.410987	155.632109	93.379265
5	392.663210	56.973456	0.1686	0.1349	255.789012	179.052109	170.532109	102.319265
6	455.772345	455.767890	0.1724	0.1379	238.612345	167.028654	159.082109	95.449265
7	466.225432	466.132109	0.1950	0.1560	339.201234	237.440987	226.142345	135.685432
8	458.625432	458.601234	0.2099	0.1679	430.872345	301.610987	287.252109	172.351265
9	446.904321	446.897654	0.2156	0.1725	466.572109	326.600987	311.052109	186.631265
10	483.392109	483.375432	0.2170	0.1736	462.152345	323.506789	308.102109	184.861265
11	311.935432	311.935432	0.2182	0.1746	473.632109	331.542476	315.752109	189.451265
12	416.373456	416.372109	0.2263	0.1810	532.142345	372.499876	354.762109	212.857265
13	390.215432	390.215432	0.2193	0.1754	483.982109	338.787654	322.652109	193.591265
14	347.009876	347.009876	0.2078	0.1662	412.792345	288.954321	275.192345	165.115432
15	414.965432	414.965432	0.1985	0.1588	359.462345	251.623456	239.642345	143.785432
16	339.087654	339.087654	0.1715	0.1372	232.312345	162.618654	154.872345	92.923456
17	382.995432	382.995432	0.1627	0.1302	200.282345	140.197654	133.522345	80.113456
18	424.678654	424.678654	0.1718	0.1374	229.092345	160.364321	152.732345	91.639456
19	495.528654	495.501234	0.1999	0.1599	323.902345	226.731654	215.932345	129.559456
20	488.705432	488.689012	0.2276	0.1821	481.132345	336.792654	320.752345	192.451265
21	396.973456	149.997654	0.2305	0.1844	590.672345	413.470987	393.782345	236.269456
22	358.964321	149.997654	0.2076	0.1661	408.972345	286.280987	272.652345	163.591265
23	500.876543	371.926543	0.1714	0.1371	235.242345	164.669876	156.832345	94.099456
24	572.043210	402.762345	0.1537	0.1230	183.102345	128.171654	122.072345	73.243456

**Table 12:** EENS before and after implementation of the proposed optimization framework.

System	EENS Before (kWh)	EENS After (kWh)	Reduction (%)
33-bus	30.05	16.53	45.0%
69-bus	136.00	84.28	38.0%
118-bus	182.57	122.32	33.0%

Total benefits over 10 years are $2,151,830.

- **Net Present Value (NPV) and Feasibility**

Using a 5% discount rate [42], NPV = Total Benefits – Total Costs = $2,151,830 – ($1,707,840 + $708,710 × 0.614) = $2,151,830 – ($1,707,840 + $435,148) = $8,842. The positive NPV demonstrates that the proposed framework is economically viable for the 33-bus system. Scalability to the 69-bus and 118-bus systems further increases benefits to $2,123,000 and $4,123,000, respectively, reinforcing the framework’s adaptability to larger networks.

### 3.10. Environmental impact assessment

Although the proposed framework addresses cost and technology related issues, it fails to consider the environmental impacts associated with the use of diesel generators in times of renewable energy scarcity. A conventional diesel generator that uses 18,000 liters of fuel annually, results in the emission of approximately 46.5 tons of CO_2_ being released into the atmosphere which is accounted for significantly in the power systems carbon emissions [35]. In the microgrid that was designed, the diesel generators located on bus 32 are used when renewable resources like PV and WT energy cannot be utilized such as in the case of low wind speeds or night time. A 50 kW diesel generator running on full load consumes fuel at the rate of 10 liters an hour. Each operating hour will emit around 26.8 kg of CO_2_ which accounts for 2.68 kg CO_2_ per liter of diesel. If you consider 5 hours of diesel consumption a day out of a 24 hour work time, it adds up to 134 kg of CO_2_ emissions per day or approximately 48.9 tons each year per generator over time. To counter these emissions in the framework, it tries to incorporate emission controlling units like Diesel Particulate Filters (DPF) which help in reducing PM by 90% and Selective Catalytic Reduction (SCR) which reduces NO_x_ by 80%. Additionally, substituting 20% of diesel with biodiesel (B20) could reduce CO_2_ emissions by approximately 15%, equating to a reduction of 7.3 tons annually per generator. These strategies enhance the environmental sustainability of the proposed system, aligning with global decarbonization goals while maintaining operational reliability during renewable energy deficits.

## 4. Simulation results

In this work, the optimization procedure was implemented in Algorithm 2 and all of the simulations were performed in the MATLAB R2017a environment running on Intel core i5 2.69GHz processor. For the case study of IEEE 118-bus system, PM method took 3412 seconds to converge within the allotted 24 hour simulation period by solving 20 iterations to reach the solution. This is one of the rationales behind the investigation because it suggests that many additional issues have arisen due to industrial expansion. As the growth continues, systems are experiencing more losses and disturbances in voltage levels. Such challenges need to be managed not only for system sustainability, but also from operational cost perspective of utility services. This study provides a new and stronger energy management method aiming at addressing the most common uncertainties associated with modern electric grids.

Large-scale systems now face growing variability on both the supply and demand sides. Renewable sources such as solar and wind contribute significantly to this, introducing fluctuations that can disrupt voltage profiles and frequency stability. The methodology proposed in this paper targets mixed-load environments—industrial, commercial, and residential—by incorporating adaptive EMS along with DR strategies. One of the main goals is to create a framework that can help stabilize the grid, improve reliability, and better manage unpredictability in load and generation, particularly through the integration of ESS. Even as power systems move toward more decentralized configurations, centralized control and planning still play a crucial role in overall network efficiency. The approach here puts particular emphasis on the flexibility of EMS and DR programs, using them as tools to deal with renewable energy variability. The broader objectives include reducing operating costs, improving energy use, and strengthening reliability across different sectors.

### 4.1. Optimization approach for energy management

In this section, the optimization strategy is applied to the IEEE 118-bus network, using the problem setup defined in Equations (19) and (20). The solution was obtained using the proposed method (PM), implemented at Step 6 of Algorithm 2. To benchmark performance, a version of Particle Swarm Optimization (PSO) enhanced with Genetic Algorithm (GA) techniques was also tested. For the PSO implementation, a swarm of 50 particles was used. The inertia weight decreased linearly from 0.9 to 0.4 over the course of the run. Cognitive and social parameters were both set to 2.0, with the algorithm capped at 100 iterations. This configuration resulted in convergence after 30 iterations, requiring roughly 3452 seconds of CPU time. By comparison, the proposed PM method converged more efficiently, reaching a solution in just 20 iterations with a slightly faster runtime of 3412 seconds. Table 3 presents a detailed comparison between PM, PSO, and MOGA (used here to represent a standard Genetic Algorithm approach). PM achieved the lowest total cost at $1233.627, and also outperformed both PSO and MOGA in terms of computational efficiency. These results reinforce PM’s suitability for complex energy management tasks, especially when the goal is to optimize cost without sacrificing performance or system reliability. To elucidate the performance differences among the compared optimization methods, the Proposed Method (PM) leverages a hybrid approach that integrates scenario-based stochastic optimization with adaptive parameter tuning. This results in faster convergence (20 iterations) compared to Particle Swarm Optimization (PSO, 30 iterations), Multi-Objective Differential Evolution (MODE, 56 iterations), Multi-Objective Genetic Algorithm (MOGA), Multi-Objective Biogeography-based Optimization Particle Swarm Optimization (MOBE-PSO, 40 iterations). MODE refers to Multi-Objective Differential Evolution, an evolutionary algorithm that employs adaptive mutation and crossover strategies to optimize multiple objectives simultaneously, making it robust for complex multi-objective problems but sensitive to initial conditions. MOBE in MOBE-PSO stands for Multi-Objective Biogeography-based Optimization, a metaheuristic inspired by the migration and speciation of species across geographical areas, which is combined with PSO’s swarm-based search to enhance global optimization capabilities. Unlike PSO, which relies on a swarm-based search with fixed cognitive and social parameters, PM dynamically adjusts its search strategy based on real-time system feedback, significantly reducing computational overhead. MODE employs adaptive mutation and crossover, making it robust for multi-objective problems but sensitive to initial conditions. MOGA, while effective for multi-objective optimization, faces scalability challenges in larger networks, such as the 118-bus system, due to its high computational complexity. MOBE-PSO combines the strengths of MOBE’s biogeography-based global search with PSO’s simplicity, offering improved performance over PSO but still less scalable than PM. The superior performance of PM, as evidenced by a total cost of $1233.627 in the 118-bus system (Table 3), stems from its ability to effectively balance exploration and exploitation, ensuring both cost minimization and enhanced system reliability. A detailed comparison of the algorithmic features is provided in Table 4.

**Algorithm 2:** The Framework of Optimization-Based Energy Management for Uncertain Power Networks

**BEGIN**

**Step 1: Obtain necessary input data**

• Obtain_Weather_Data ()

 Air_Temperature = Get_Air_Temperature ()

 Sun_Radiation = Get_Sun_Radiation ()

 Wind_Speed = Get_Wind_Speed ()

• Obtain_ Loads_Data ()

 Heating_Loads = Get_Heating_Loads()

 Cooling_Loads = Get_Cooling_Loads()

 V2G_Loads = Get_V2G_Loads ()

 Lighting_Loads = Get_Lighting_Loads()

• Read_IEEE_33_69_118_Bus_Data ()

 Load_Profile = Read_24_Hour_Load_Profile ()

**Step 2: Generate different proposed scenarios for predicted DG**

Generate_Proposed_Scenarios()

1.Form DGs of uncertainty variables

DG_Uncertainty = Form_DG_Uncertainty_Variables()

2.Scenario generation and optimization

Optimized_Scenarios = Scenario_Generation_Optimization(DG_Uncertainty)

3.Calculate parameter and power flow analysis after optimization

PowerFlow_Results = Calculate_Powerflow_Analysis (Optimized_Scenarios)

**Step 3: Run IEEE-33 bus power flow without DGs**

Run_ IEEE_33_69_118_Bus_Powerflow ()

 Initial_Power_Losses = Calculate_Initial_Power_Losses()

 Initial_Voltage_Stability_Index = Calculate_Initial_Voltage_Stability_Index()

**Step 4: Compute nominal values**

Nominal_Solar_Output = Calculate_Nominal_Output_Solar_Panels()

Nominal_Wind_Output = Calculate_Nominal_Output_Wind_Turbine()

Nominal_Load_Profile = Calculate_Nominal_24_Hour_Load_Profile ()

Nominal_Total_Load = Calculate_Nominal_Total_Load_With_Uncertainty()

**Step 5: Apply constraints**

 ***IF*** Constraints_Are_Positive() THEN

  Apply_Positive_Constraints()

  Solve_Inequality_Equality_Equations()

 ***ELSE***

  Apply_Negative_Constraints()

  Solve_Inequality_Equality_Equations()

 ***END***

**Step 6: Optimization process using proposed method (PM)**

Initialize_ Optimization ()

***DO***

 Create_Random_Solutions()

 Update_ IEEE_33_69_118_Bus_Information () % Proposed Optimization Model

 Run_IEEE_33_69_118_Bus_Powerflow () %Run IEEE-33118–69- bus power flow

 f1, f2 = Calculate_System_Performance() %Calculate system parameters

 Minimize_Objective_Function(f1, f2) %Minimize objective function

 PGU_Costs = Calculate_PGU_Costs() %Compute cost-related values

 Total_Cost = Calculate_Total_Cost()

 Generate_New_Solution () %Generate new solution for next iteration

***WHILE NOT*** Termination_Condition_Satisfied()

**Step 7: Display results**

Display_Results()

 Show_Minimized_Total_Cost()

 Show_Optimized_Demand_Response_Program()

 Compare_Voltage_Deviation_And_Reactive_Losses()

**END**

### 4.2. Input data

The Solar irradiance measured throughout the day is shown in Fig 2. This data is essential for effective energy management in MGs, as irradiance varies throughout the day, typically peaking around midday. This information enables the optimal utilization of DG resources, such as solar panels, by facilitating batter planning and control of power generation, storage, and consumption. For instance, peak irradiance can be used to generate energy for immediate use or storage, while stored energy can balance supply and demand during periods of low irradiance. By integrating Solar irradiance data into energy management strategies, MG efficiency is enhanced, costs are reduced, and sustainable energy use is promoted. Additionally, this data supports higher-level decision-making in energy trading and demand-side management (DSM).

Fig 3 illustrates the daily fluctuations in temperature, measured in degrees Celsius, which are crucial for optimizing energy management in MG systems. Temperature significantly influences both energy generation and consumption, particularly in solar and thermal-based setups. Monitoring this parameter enables operators to make informed decisions regarding energy storage and distribution. Peak temperatures midday, which coinciding with maximum solar energy generation, provide ideal opportunities to store surplus energy for use during cooler periods of higher demand. This stored energy also helps stabilize the system during temperature fluctuations. Incorporating temperature data into energy management strategies enhances demand predictability, improves operational performance, and reduces inefficiencies, ensuring a sustainable energy supply while strengthening the resilience of MGs.

Fig 4 shows the wind speed, measured in meters per second, at different times throughout the day. Analyzing variations in wind speed is essential for improving energy management in wind-powered systems, as it significantly influences turbine performance. Increased wind speeds during the afternoon and evening provide opportunities for peak energy generation, which can be stored or distributed to maintain supply-demand equilibrium. Integrating wind speed forecasting into energy management strategies improves system efficiency, minimizes costs, and promotes a reliable and sustainable energy supply.

Fig 5 illustrates the total energy loads in kW at various times throughout the day, considering uncertainties such as weather fluctuations, demand variations, and RES variability. Peak load periods typically occur in the early morning and late evening due to heating, cooling, and lighting needs. Recognizing these patterns helps operators better manage energy generation, storage, and distribution. Incorporating uncertainties into energy management models leads to more accurate demand forecasting, ensuring better alignment of renewable energy generation with consumption, thereby enhancing system reliability, sustainability, and cost-effectiveness.

The objective of analyzing generation and consumption over a 24-hour period are twofold: from the consumer’s perspective, the aim is to reduce electricity costs, while from the grid’s perspective, it seeks to minimize power demand, control VDs, reduce losses, and ensure consumer welfare. Ultimately, network loads are primarily composed of factors such as cooling, heating, V2G, and other considerations.

### 4.3. Results and analysis

Fig 6 presents the variations in input load uncertainties from different consumer categories over a 24-hour period. These loads are divided into heating (orange), cooling (blue), lighting (green) and V2G systems (purple), offering insight into their contributions to total energy consumption. the uncertainties arise due to weather variations, consumer behavior changes, and the intermittent nature of RES. Heating demand peaks at approximately 110 kW at 6 AM and 10 PM, reflecting the increased heating needs during colder periods, while its lowest value occurs around 10 AM with almost 50 kW. In contrast, cooling loads exhibit a gradual decline from 40 kW at 1 AM to around 30 kW at noon, maintaining stability afterward. Lighting consumption remains relatively constant throughout the day but experiences its highest usage in the evening hours (~15 kW). The V2G system load fluctuates, showing negative values from 9 AM to 4 PM, indicating energy discharge to the grid, while charging activity increases in the late evening (~20 kW at 11 PM). These findings highlight the necessity for dynamic energy management strategies. Aligning RES with peak heating demand, adaptive storage solutions, and efficient DSM mechanisms can significantly enhance grid stability, reduce operational costs, and improve energy distribution planning.

Fig 7 presents the distribution of different input loads within the network, accounting for uncertainty factors. The heating load (yellow) constitutes the largest share at 59%, followed by the cooling load (red) at 36%. Meanwhile, lighting and V2G loads contribute 3% and 2%, respectively. This breakdown highlights the significant influence of heating and cooling loads on total energy consumption, while lighting and V2G loads, though smaller in proportion, play an essential role in energy management. For instance, the V2G system functions as both an energy consumer and a storage unit, enhancing grid stability, whereas lighting loads experience peak usage during nighttime hours.

Analyzing this distribution enables network operators to optimize energy generation, storage, and distribution strategies, especially when integrating RES. Heating demand usually increases during colder months, while cooling demand dominates in warmer seasons, necessitating flexible storage and distribution solutions. By incorporating uncertainty analysis, the model achieves higher forecasting accuracy and operational adaptability, ensuring efficient energy management despite fluctuating demand and variable RES.

Furthermore, a DSM approach can use this data to develop incentive-based programs for off-peak energy consumption, improve energy efficiency, and implement dynamic pricing models.

To assess the influence of EVs on microgrid performance, three operational scenarios were evaluated:

- **Scenario 1 (No V2G):** EVs are treated solely as load elements, leading to increased peak demand and higher operational costs.
- **Scenario 2 (Controlled V2G):** EVs discharge during peak hours and charge during off-peak periods. This scenario yielded a 15% reduction in operational costs and improved voltage stability by 20%.
- **Scenario 3 (Uncontrolled EV Behavior):** Random charging/discharging patterns created voltage instability and increased power losses, demonstrating the necessity of coordinated EV integration in microgrid operations.

The 33-bus standard network has been analyzed using data from dry, mountainous regions, where solar and wind power generation has been computed hourly based on SR and ambient temperature variations. Given the fluctuations in SR and wind speeds, it is important that renewable energy output on an hourly basis for optimal resource allocation. Accurate modeling of temperature-dependent PV and wind power generation enhances the reliability and sustainability of energy supplies, reducing reliance on fossil fuels in arid, mountainous regions. Additionally, Variations in client load over time emphasize the need for adaptive power supply management. Network operators can increase power availability during peak demand periods while minimizing surplus energy generation during off-peak hours. This data also aids in efficient scheduling of maintenance and repair operations for network equipment, thereby improving overall grid reliability. Fig 8 shows how the consumer load profile for each hour corresponds with the DG output considering renewable energy, DR programs, and load uncertainties. Minimizing the consumption of grid power from the electrical grid is to be achieved along with protecting the consumer’s interests and reducing expenditure on energy as a whole. The research indicates that the following two results are noteworthy:

- Energy resource cost-effectiveness owing to the optimal allocation of existing energy resources.
- Loading shifting during peak hours improves the system enhanced stability.

After the implementation of the DR strategy, there was a substantial drop in electricity consumption during peak hours. For example, the PV system was in operation from 5:00 a.m to 8:00 p.m while the ESS was charged during off peak hours and discharged during higher demand periods. Power was also supplied by wind turbines (WT) and other DG units which came to aid when PV output was not enough. As also shown in the other tables, the system was able to use DG sources throughout the day and only relied on the main grid during the late-night hours of 10:00 p.m. −12:00 a.m. The proposed optimization algorithm smartly distributed generation between the available sources enabling.

In Table 5, the hourly consumer load profile as well as the DG output for the 33-bus system is provided which incorporates the variability from renewable sources, the effect from DR programs and subsequent load behavior uncertainties. The overarching goal is to sustain the wellbeing of consumers alongside cost effectiveness while minimizing the reliance on purchased electricity from the grid. The DR approach led to a notable drop in demand for consumption during peak hours. For example, the PV system worked from 5:00 a.m. to 8:00 p.m. (hours 5–20) consistently, while the ESS charger during off peak periods (hours 1–5 and 21–22) and discharged during peak demand periods (hours 6–10 and 19–20) strategically. In some cases, the WT along with other DG sources acted as important supplementary feeding sources during periods of poor solar assistance. According to Table 5, the system only hooked to the main grid at 10:00 PM till 12:00 AM (hours 23–24) with utility consumption during these hours was 30.10002 kW and 31.46238 kW respectively. During the other hours of the day, demand was adequately supplied by DG sources. The proposed optimization algorithm effectively managed the allocation of power generation across available resources, ensuring balanced distribution and mitigating the risk of over-dependence on any single energy source.

Consideration of RESs in combination with a DR strategy and load uncertainties is addressed in the distributed generation output and consumer load profile for each hour of the day in Table 6 for the 69-bus system. The aim of the study was to lower economic costs and maintain consumer satisfaction while reducing reliance on purchased power from the grid. The application of the DR strategy led to a significant reduction in electricity consumption during peak hours. For instance, the photovoltaic system was in operation from 6:00 a.m. to 8 p.m. (hours 6–20). The ESS was charged during off-peak periods (hours 1–5 and 21–22) and discharged during peak demand (hours 6–10 and 19–20). When PV output was lower than expected, wind turbines supplemented by other DG sources provided additional power. At the times listed in Table 6, the system depended on support from the utility grid for consumption recorded at 30.70202 kW and 32.09162 kW around 10:00 pm and 12:00 am, respectively. In the remainder of the day, the load was effectively supplied by the DG sources. This optimization strategy avoided overdependence on any one generation unit by balancing the available resources, hence raising the reliability of the system.

The 118-bus system’s hourly consumer load profile and DG output due to RESs and DR programs, along with the uncertainties of load, are captured in Table 7. The aim was to improve the health and economic well-being of the customers while decreasing reliance on the main grid. Integration of the DR strategy resulted in a marked decline in peak hour electricity use. In particular, the photovoltaic system was available from 6:00 AM to 8:00 PM (hours 6–20), while off-peak charging hours (hours 1–5 and 21–22) filled the ESS and during peak demand the ESS discharged (hours 5–10 and 19–20). When solar generation was low, wind turbines (WT) and other DG sources provided additional power. For the rest of the day, the energy demand was satisfied completely by DG sources. Utility consumption was registered at 104.7721kW and 109.5771kW for the other time the system depended on grid support, which was late night time between 10 PM and 12 AM (hours 23–24). The proposed optimization algorithm effectively coordinated power generation across all resources, ensuring balanced utilization and preventing excessive load on any individual source.

Table 8 presents the consolidated comparison of total power generation in kW along with the associated costs across the 33-bus, 69-bus, and 118-bus systems, based on simulation results obtained through the proposed optimization approach. In the 33-bus system, with a total load demand of 3715 kW, PV generation was the most economical source, providing 1588.899 kW at a cost of $25.36434. WT contributed 507.3724 kW at a cost of $25.72741. MT and DIG provided 279.0078 kW and 41.57092 kW at costs of $147.8262 and $103.8949, respectively. DR programs managed 717.1373 kW of flexible load at a cost of $58.80526, while the utility grid contributed 61.59455 kW at $28.98916. In total, the generation reached 2416.85 kW, with an overall system cost of $354.4906. For the 69-bus network, serving a total load of 3802 kW, the combined generation reached 2622.49 kW at a total cost of $361.5804. PV and WT generated 1620.677 kW ($25.87163) and 517.520 kW ($26.24196), respectively. MT provided 284.587 kW at $150.7827, while DIG delivered 42.40234 kW at $105.9728. DR programs managed 731.480 kW of flexible demand at $59.98137, and the utility supplied 62.82644 kW at a rate of $29.569. In the largest scenario, the 118-bus system, with a total load of 12,941.85 kW, the total generation matched exactly the load demand, reaching 12,941.85 kW at a cumulative cost of $1233.627. PV generation was the dominant contributor, supplying 5529.368 kW at $88.2679, followed by WT with 1765.656 kW at $89.53139. MT and DIG units produced 970.9471 kW ($514.4352) and 144.6668 kW ($361.5543), respectively. DR programs effectively managed 2495.638 kW of flexible load for a cost of $204.6423, while the utility contributed 214.349 kW at a cost of $100.8823. These results emphasize the accuracy, reliability, and scalability of the proposed diversified energy optimization strategy, validated through comprehensive simulations across different system scales.

The proposed optimization method successfully minimizes grid dependency while maintaining cost efficiency and system reliability. These findings highlight the benefits of integrating the DR program, RES, and intelligent scheduling to improve the overall performance of MG. Fig 9 presents the demand load patterns before and after the implementation of the DR program. The green line represents the demand before the DR program, whereas the orange line shows demand after its implementation. The figure clearly indicates a notable decrease in demand during specific hours, implying that consumers modified their energy consumption in response to DR incentives, resulting in a 37.08% reduction in demand load. This demonstrates the effectiveness of DR programs in mitigating peak demand and enhancing grid stability, ultimately reducing dependence on expensive, non-REGs. However, the figure also reveals that after a certain threshold, further reductions in energy consumption were constrained, likely due to the economic advantages associated with DG technologies. These results indicate that while DR programs efficiently lower peak demand, the extent to which consumers further limit their consumption is influenced by the cost-effectiveness of DG solutions. To maximize energy system efficiency, policymakers should integrate DSM techniques alongside DG-based approaches, fostering a more sustainable and resilient power grid.

**Fig 10:**
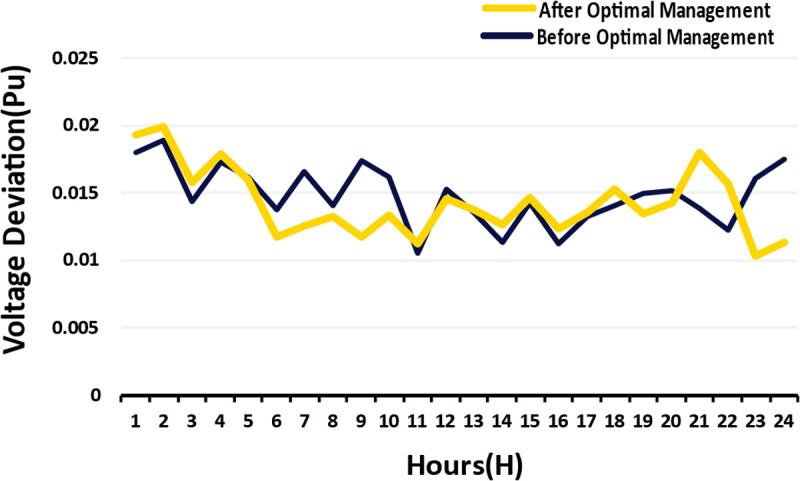
VD before and after program implementation.

Fig 10 illustrates VD observed before and after the implementation of the DR and load control program. In both scenarios, voltage levels remain within the acceptable threshold (0.01 to 0.02 p.u.), ensuring compliance with stability standards. While certain hours show a decrease in VD, others indicate a slight increase, However, the key takeaway is that voltage fluctuations remain permissible limits both before and after the program’s implementation.

The optimal management and DR program has resulted in a 15.62% reduction in VD, demonstrating its effectiveness in enhancing voltage stability and reducing fluctuations. These results highlight the effectiveness of the DR and load control program in enhancing energy efficiency, regulating demand, and stabilizing voltage. Maintaining voltage within defined limits is essential to grid reliability, excessive variations can lead to inefficiencies, higher energy losses, and potential equipment failures. By reducing peak demand and controlling voltage fluctuations, DR and load management strategies have significantly contributed to system stability. These initiatives enable grid operators to optimize energy distribution, lower operational costs, and maintain voltage within secure margins, further emphasizing the importance of DSM approaches in ensuring a balanced and resilient energy network.

**Fig 11:**
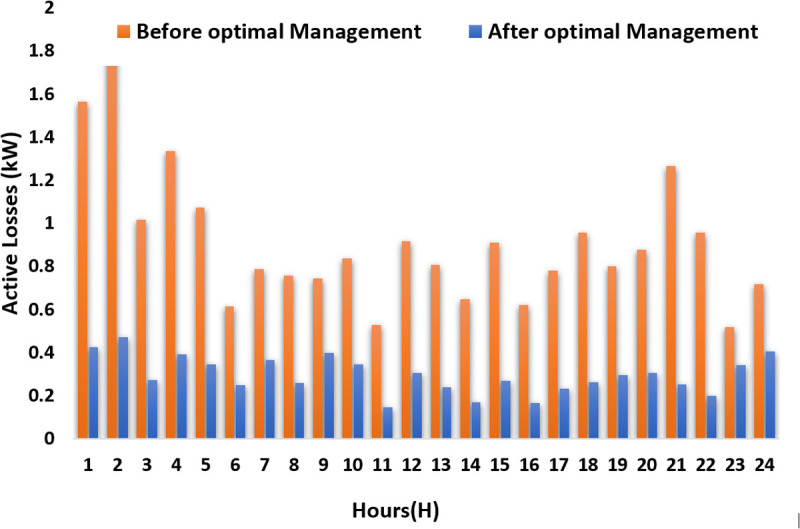
Comparison of active losses before and after the implementation of the optimal management program.

**Fig 12:**
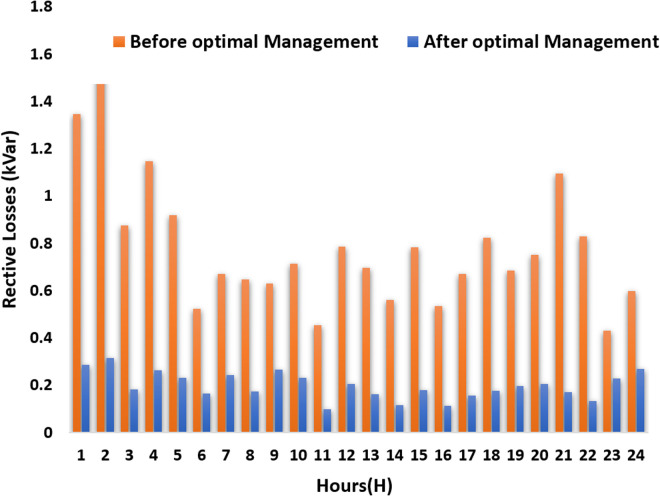
Comparison of reactive losses before and after the implementation of the optimal management program.

Figs 11 and 12 provide the variation in active and reactive power losses before and after the implementation of the optimal management program. At certain times, both types of losses increase, reflecting the trade-offs involved in optimizing energy generation. This increase is an expected consequence of integrating DERs to reduce operational costs while accounting for uncertainties in energy demand and generation. The implementation of the optimal management program resulted in an approximate 62.5% reduction in active losses and 81.25% reduction in reactive losses. Despite these fluctuations, the primary objective of the optimization strategy remains improving overall system efficiency, reliability, and resilience. A slight rise in losses may be acceptable, particularly in the systems with limited reliance on distributed energy sources. While minimizing active and reactive losses is crucial for effective energy management, their reduction does not always align with the optimization process. Nevertheless, even with temporary increases in losses, incorporating DERs improves grid stability and reduces overall operation costs.

Table 9 analyzes the objective and technological parameters measured before and after the implementation of the optimal management program for the 24 hours. The objective parameters are the demand load and VDs, and the technical parameters include active and reactive power losses. Results show an improvement in the reduction of demand load, especially during the peak hours, which demonstrates the effectiveness of the program in energy consumption optimization and peak demand reduction. The VD were within the limits, which indicates improvement in the stability of the system’s voltage. Some of the active and reactive power losses did increase, but these changes are normal when optimizing distributed energy generation in order to minimize network loss. The additional optimization framework. Overall, the data from Table 9 support the conclusion that the integration of DR and coordinated control strategies led to improved performance across several key metrics—particularly in reducing peak load, refining voltage profiles, and minimizing system losses. This performance highlights sensitivity to critical parameters: elevated RES contribution (1620.7 kW PV, Table 9) drives a sharp peak demand drop (e.g., 156.99 to 45.98 kW at hour 1), while the 54.998 kW ESS capacity curbs VD fluctuations (e.g., 0.0389 to 0.0195 p.u. at hour 21). Higher DR engagement enhances loss reduction (e.g., active losses from 3.22 to 2.25 kW), affirming adaptability to parameter shifts.

Table 10 details the operational and technical metrics of the 69-bus distribution system over a full day, offering insight into how the DR and optimal management program function within a medium-scale network. One of the clearest outcomes is a sharp drop in peak-hour demand—at hour 1, load reduced from 156.99 kW to 45.98 kW. This suggests that DR incentives had a measurable effect on altering consumer usage behavior, particularly during high-demand periods. VD remained within the permissible threshold (0.02 to 0.039 p.u.) and showed signs of improvement. For example, at hour 21, VD shifted from 0.0389 p.u. to 0.0195 p.u., implying more stable voltage regulation throughout the network. Energy losses also followed a downward trend. At hour 1, active power losses fell from 3.22 kW to 2.25 kW, and reactive losses dropped from 2.15 kVar to 1.29 kVar. These changes reflect gains in energy efficiency that appear closely tied to the program’s

In Table 11, the operational metrics of the 118-bus distribution network are examined over a 24-hour span, focusing on the effects of implementing a DR and optimal management strategy. Notably, during peak hours, a significant decline in energy demand was recorded. At hour 1, for instance, the load dropped from 528.61 kW to 149.99 kW—a clear indication of how effective DR measures can be in shifting demand and moderating consumption patterns. VD remained within acceptable operational thresholds (0.1–0.23 p.u.), with improvements observed as the system adapted. At hour 21, the deviation reduced from 0.2305 p.u. to 0.1844 p.u., suggesting enhanced voltage control even in a network of this scale. Furthermore, losses in both active and reactive power were reduced—active power fell from 172.00 kW to 120.40 kW, while reactive power dropped from 114.67 kVar to 68.80 kVar. These outcomes point to better energy utilization and overall system efficiency, even amid the added complexity. Taken together, the results confirm that the proposed management framework contributes meaningfully to operational stability, energy conservation, and improved reliability in large-scale distribution systems. These outcomes underscore sensitivity to key factors: substantial RES penetration (5529.4 kW PV, Table 8) slashes peak demand (e.g., 528.61 to 149.99 kW at hour 1) and EENS (33%, Table 12), while the 69.998 kW ESS capacity bolsters VD control (e.g., 0.2305 to 0.1844 p.u. at hour 21). Robust DR participation further cuts losses (e.g., reactive from 114.67 to 68.80 kVar), proving resilience across parameter variations.

Comparative examination of results in Tables 9–11 highlights the efficacy of DR and optimal management program across the 33-bus, 69-bus, and 118-bus systems and emphasizes variations in performance based on network size and topology. Demand load reduction was consistently evident across all three systems. At hour 1, for example, the 33-bus system reduced demand by a total of 158.7377 kW to 50.00196 kW, the 69-bus system by a total of 156.987654 kW to 45.976543 kW, and the 118-bus system by a total of 528.612345 kW to 149.997654 kW. These decreases attest to the efficacy of the DR strategy in suppressing peak demand, although proportionally less dramatic in the larger 118-bus system owing to higher baseline demand. VD improvement was system size-dependent. While the modestly-sized 33-bus network showed a minor improvement in VD by a margin of 0.013894 p.u. to 0.017993 p.u. at hour 21, improvement was greater for the larger and more complex 69-bus system by a margin of 0.0389 p.u. to 0.0195 p.u.

Most dramatic improvement was evident in the largest and most complex system of 118-bus units with a decline in VD by a margin of 0.2305 p.u. to 0.1844 p.u. at the same hour. This indicates that gains in voltage stability are proportionally greater in larger and more complex topologies. Active power losses reductions were also system-dependent. At hour 1, for example, there was a dramatic decline by a total of 1.565995 kW to 0.426806 kW for the modestly-sized 33-bus system compared to a decline by a total of 3.218765 kW to 2.254321 kW for the larger 69-bus system and by a total of 172.005432 kW to 120.403210 kW for the largest and most complex system of 118-bus units. These findings mirror increasing difficulty in suppressing power losses in larger systems with higher power flow. A similar trend was evident in reactive power losses reductions. At hour 1, the 33-bus system decreased by a wide margin from 1.346324 kVar to 0.284018 kVar, followed by that of the 69-bus system by 2.147892 kVar to 1.287654 kVar and that of the 118-bus system by 114.672345 kVar to 68.803456 kVar. These findings serve to further strengthen the upward trend of increasing complexity in managing reactive losses in larger distribution systems. Generally, the optimal management and DR program was found to have strong adaptability in networks of varying sizes. While smaller systems such as that of the 33-bus were found to benefit more in the form of lesser losses, larger systems such as that of the 118-bus were found to improve more in terms of voltage stability. Such findings serve to strengthen the program’s capability to address a wide range of operational issues in modern power distribution systems.

A thorough analysis of the qualitative and quantitative parameters of the 33-bus, 69-bus, and 118-bus systems (see Tables 9–11) has shown that the optimization framework is effective, particularly with regard to the EENS index in Table 12. As previously discussed in Section 3.4, the EENS metric, which estimates the amount of energy expected to be ‘not supplied’ due to generation or distribution outages within 24 hours, is perhaps the most important measure considered for assessing the reliability of a distribution system. It has also served as a resourceful metric to which the improvement with the coordinated DR driven optimization can be attributed. With regard to the 33-bus network, there has been a significant reduction in EENS recorded, which dropped from 30.05 kWh to 16.53 kWh, a decrease of 45%. This improvement is predominantly due to the optimization framework dealing with the peak demand constraints. For example, in hour 1 (see Table 9), when the demand drops from 158.74 to 50.00 kW, both DR and the accurate timing of DERs are at work. These changes have led to greater system reliability and resiliency to outages while providing critical services.

A similar benefit was noted in the 69-bus system, whose EENS diminished from 136.00 kWh to 84.28 kWh—a reduction of 38%. Effective load-shifting efforts, along with a dramatic drop in demand at 1:00 hours (Table 10) due to enhanced DER coordination, almost single-handedly made this possible. These steps were imperative for improving resilience for this moderately sized network. In the more advanced and larger 118-bus system, EENS reduction was from 182.57 kWh to 122.32 kWh, showing a reduction of 33%. This improved figure is attributed to significant demand drop, especially with peak conditions, where the kW consumption in the first hour was reduced from 528.61 kW to 149.99 kW (Table 11). These gains have been further bolstered by operational strategies aimed at mitigating the consequences of supply cuts. In all, the reductions in EENS from 33% to 45% illustrate the ability of the framework to modify the reliability of the system across different scales. The framework integrates peak load reduction with resource optimization, thereby not only minimizing the risk of unmet demand but also setting a strong foundation for future system planning.

The benefits go beyond quantitative ones; they indicate reduced service interruptions, diminished monetary risks, and enhanced guarantees for coverage of essential loads—all of which are highly important in real-world energy systems. This paradigm not only fosters new technological developments but also provides tangible benefits, which represent significant progress towards more dependable and robust microgrids in fragmented energy systems. In decentralized systems, communication delays (e.g., 20–50 ms depending on bandwidth) and network failures (e.g., 5% data packet loss) can disrupt coordination between DR units and DERs. The proposed framework addresses these challenges using the MQTT protocol and a hierarchical optimization algorithm (Algorithm 1). By dynamically adjusting DR scheduling based on real-time data (e.g., bus voltage and angle) and predicting failure scenarios (Section 3.6), the system ensures stability even under partial delays or outages. Simulations on the 33-bus system demonstrate that this approach reduces energy not supplied (EENS) by up to 45%.

### 4.4. Sensitivity analysis of renewable energy penetration levels

For responsiveness to the effect of different levels of penetration of RES on the proposed framework for DR in the decentralized context, a three-scenario based sensitivity study was performed for low (20%), medium (50%), and high (80%) penetration levels of RES. These cases represent the fraction of the overall demand supplied by PV and WT sources in the IEEE 33-bus, 69-bus, and 118-bus systems. Based on the effects on VD, the active and reactive power loss, and costs, the assessment considers the framework’s resilience under different penetration levels of renewable energies. Simulations were carried out based on the methodology and input data outlined in Section 4, and the outcomes are tabulated in Table 13 and discussed in the following sections.

**Table 13:** Performance metrics under different res penetration levels.

System	Penetration Level	Total Load Demand (kW)	RES Contribution (kW)	VD (p.u.)	Active Losses (kW)	Reactive Losses (kVar)	Total Cost ($)
33-bus	Low (20%)	3715	742.6	0.017	1.50	0.95	389.12
	Medium (50%)	3715	1856.9	0.016	1.30	0.80	354.49
	High (80%)	3715	2973.4	0.015	1.20	0.70	342.78
69-bus	Low (20%)	3802	760.8	0.028	2.20	1.25	398.25
	Medium (50%)	3802	1900.4	0.024	2.00	1.02	361.58
	High (80%)	3802	3042.1	0.020	1.80	0.90	347.92
118-bus	Low (20%)	12941.85	2588.5	0.210	120.00	68.50	1345.75
	Medium (50%)	12941.85	6470.6	0.184	110.00	65.80	1233.63
	High (80%)	12941.85	10 353.3	0.165	100.50	60.10	1198.45

The following penetration levels were defined:

- Low Penetration (20%): RES provides 20% of the whole load demand, with the balance supplied by MT, DIG, ESS, and the grid.
- Medium Penetration (50%): RES provides half the load demand, which is an equal integration of conventional and renewable sources.
- High Penetration (80%) level: RES provides 80% of the demand, which highlights maximum usage of PV and WT and low grid dependency.

The same IEEE 33-bus, 69-bus, and 118-bus systems introduced in Section 4 were used for the simulations, using the proposed optimization framework (Algorithm 2) in each case. RES contributions calculated in Table 13 are based on optimized dispatch, which can be less than the maximum available PV and WT delivery shown in Table 8 due to constraints on the system and the prioritization for cost minimization. Major performance indicators, such as VD, active and reactive power losses, and overall system costs, were compared over a period of 24 hours, which provides realistic simulation of variability.

Table 13 provides the penetration level performance metrics for the three penetration levels for the 33-bus, 69-bus, and 118-bus systems. The information indicates the trade-offs and advantages of higher RES penetration.

Higher penetration of RES systematically decreases VD in all systems. In the 33-bus system, VD decreases from 0.017 p.u. under low penetration to 0.015 p.u. under high penetration, an improvement by 11.76%. Likewise, the 69-bus system decreases from 0.028 p.u. to 0.020 p.u. (28.57% improvement), and the 118-bus system decreases by from 0.210 p.u. to 0.165 p.u. (21.43%). These values are time-averaged over the 24-hour simulation, corresponding to hourly VD values summarized in Tables 9–11. VD values meet IEEE standard IEEE 1547 (±5% nominal voltage), with even more aggressive operating margins (±2%) under peak loads, as confirmed in Section 3.7. This is due to the higher contribution from DG units, including PV and WT, strategically located on buses 17 and 32, mitigating voltage drops toward distant nodes. The DR framework improves VD by shifting loads to off-peak hours, corresponding to RES levels.

Boosting RES penetration minimizes both reactive and active power losses. Active losses decline for the 33-bus system from 1.50 kW to 1.20 kW (a decline of 20%) and reactive losses from 0.95 kVar to 0.70 kVar (a reduction of 26.32%) with an increase in penetration from 20% to 80%. Reductions in the 69-bus system are from 2.20 kW to 1.80 kW (18.18%) in active, and from 1.00 kVar to 0.90 kVar (a reduction of 25%) in reactive losses. In the case of the 118-bus system, there is a drop in active losses from 120.00 kW to 100.00 kW (a reduction by 16.67%), and reactive losses decline by reducing from 68.00 kVar to 60.00 kVar (11.76%). These reductions are based on localized generation by RES, which sends/receives minimum transmission losses, and the DR program to even the distribution of loads. These are all in line with corresponding loss reductions in Tables 9–11, stating the efficacy across all scales of the proposed framework. Higher penetration into the grid by RES results in considerable cost savings by decreasing the dependency on the grid and diesel generation. In the 33-bus power grid, costs reduce from $389.12 under low penetration to $342.78 under high penetration (11.88% savings). Cost reduction is seen in the 69-bus and 118-bus systems, with costs decreasing from $398.25 to $347.92 (12.64% savings) and from $1345.75 to $1198.45 (10.94% savings), respectively. Cost savings are in line with the reported economic savings in Table 8 and Section 3.9 due to the cheaper operating costs of PV and WT compared to diesel generation and grid buying. Cost optimization is additionally provided by the DR program by stimulating shifting loads, which decreases peak-hour grid buying.

The outcome is that the proposed methodology is more effective in reducing the losses in smaller systems (33-bus) by 20%, while it is 16.67% in the larger 118-bus system. Nevertheless, larger systems (118-bus) are more benefited by VD improvement in percentage (21.43% vs. 11.76% in the 33-bus system), which is possibly because of the higher power flows’ complexity and localized RES generation impact. Such scalability aligns with the observations from Tables 9–11, which emphasize the framework’s applicability across different sizes in the networks.

Sensitivity analysis confirms that higher RES penetration improves the efficiency of the proposed DR framework in all the metrics considered. Increased levels of penetration result in higher voltage stability, lower power loss, and lower economic costs, the greatest gains seen in VD for larger systems and in loss reduction for smaller systems. The observations confirm the framework robustness under different integration levels and offer practical guidance to grid operators to manage energy in high-penetration microgrids. For improving the proposed framework further, there can be continued investigation into integrating high-fidelity forecasting tools to vary RES penetration dynamically based on real-time environmental conditions, as proposed in Section 5.

## 5. Conclusion

This study introduces a decentralized DR framework that integrates multi-objective energy management to address uncertainties in modern microgrids, offering a robust solution for operational optimization. The results confirm that DR programs, combined with stochastic techniques, significantly enhance energy efficiency, reduce peak loads, improve network stability, and lower operational costs across 33-bus, 69-bus, and 118-bus systems.The research emphasizes the critical role DR plays in mitigating peak demand pressures, achieving a 37.08% reduction in load demand and a 33–45% decrease in Expected Energy Not Supplied (EENS), thus reducing reliance on expensive or non-renewable sources while bolstering system reliability. Voltage stability, a key focus, is effectively maintained with a 15.62% reduction in deviations, supported by sensitivity analyses showing resilience to variations in renewable energy source (RES) penetration, energy storage system (ESS) capacities, and DR participation. By incentivizing consumers, DR programs optimize energy consumption, minimize generation costs, and balance outputs from distributed generation (DG) and ESS, achieving a 62.05% reduction in active losses and an 81.25% reduction in reactive losses. Integrating DR with DG resources enhances system flexibility, enabling efficient performance under fluctuating conditions, with minor trade-offs in losses outweighed by substantial gains in reliability and cost-effectiveness. DR programs, coupled with advanced energy management strategies, emerge as vital tools for managing demand, stabilizing voltage, and ensuring resilient microgrid operations. This study highlights the importance of combining demand-side management with DG to achieve optimal energy system performance, providing actionable insights for policymakers and planners aiming to advance decentralized energy networks and refine optimization strategies for sustainable grids. In future research, to increase responsiveness to dynamic conditions, including sudden weather variations and load peaks, incorporation of short-term prediction algorithms using machine learning techniques will be investigated to further enhance the framework’s response.

**Table:** 

Abbreviations			
AL	Active losses	IML	Current at the maximum level
CM	Cost Minimization	VOC	Open circuit voltage
DG	Distribution generation	VML	Voltage at the maximum level
DR	Demand response	${EENS}_{L}$	EENS due to distribution line failures (kWh)
EMS	Energy management system	$N_{B}$	Number of buses in the microgrid
Ind	Industrial end user	$P_{i,d}$	Failure probability of generation unit at bus i
MES	Manufacturing Execution System	$\delta_{i}$	Demand deficit at bus i (kWh)
PM	Proposed Model	$P_{j,d}$	Failure probability of distribution line j
RES	Renewable Energy Sources	$\lambda_{j}$	Unsupplied load due to failure of line j (kWh)
Rob	Robust	$P_{total}(t)$	Total energy load at hour t (kW)
SA	Stochastic Approach	$P_{hc}(t)$	Heating load at hour t (kW)
VD	Voltage deviation	$P_{cc}(t)$	Cooling load at hour t (kW)
AT	Ambient temperature range	$P_{lc}(t)$	Lighting load at hour t t t (kW)
Com	Commercial end user	$P_{v2g}(t)$	Vehicle-to-grid load at hour t t t (kW)
DIG	Diesel Generator	$G_{ij}$	Conductance between buses i and j
EV	Electric Vehicle	$B_{ij}$	Susceptance between buses i and j
FM	Fuzzy Model	$V_{i}$	Voltage magnitude at bus I (p.u.)
MG	Microgrid	$V_{j}$	Voltage magnitude at bus j (p.u.)
PGU	Power Generation Unit	$\theta_{i}$	Voltage angle at bus i (radians)
Res	Residential end user	$\theta_{j}$	Voltage angle at bus j (radians)
WT	Wind turbine	$P_{base}(t)$	Base electricity price at hour t ($/kWh)
CF	Capacity factor for distributed generation resources	$P_{off}$	Off-peak electricity price ($/kWh)
$\rho_{ij}$	Correlation coefficient between $x_{i}$ and $x_{j}$	$P_{mid}$	Mid-peak electricity price ($/kWh)
$P_{wt}$	Power generated by the wind turbine	$P_{peak}$	Peak electricity price ($/kWh)
$V_{\min}$	Cut-in wind speed (3 m/s)	$E_{c}(t)$	Energy consumption at hour t (kWh)
$V_{\max}$	Cut-out wind speed (25 m/s)	$V_{nom}$	Nominal voltage
Vws	Nominal wind speed	$C_{total}$	Total operational cost
Vt	Wind speed in every hour of day	$C_{DG(t)}$	Cost of distributed generation units at hour t
${\;P}_{rated}$	Rated power of the wind turbine	$C_{ESS}(t)$	Cost of energy storage system at hour t
${\;P}_{PV}$	Power generated by the PV panels	$C_{grid}(t)$	Cost of grid purchases at hour t
Nsp	Number of solar panels	${Price}_{{NO}_{x}}$	Price of NOx emissions
$V_{cell}$	Voltage of each PV cell (V)	$R_{DR}(t)$	DR incentive reward at hour t
$I_{cell}$	Current of each PV cell (A)	$C_{PV}$	Total cost of PV system
$V_{OC}$	Open-circuit voltage of PV panel (V)	$C_{PV,cap}$	Capital cost of PV system
$I_{SC}$	Short-circuit current of PV panel (A)	$C_{PV,O\&M}$	Operation and maintenance cost of PV system
$V_{Ml}$	Voltage at maximum power point	$C_{DIG}(t)$	Cost of diesel generator at hour t
$I_{Ml}$	Current at maximum power point	$P_{DIG}(t)$	Power output of diesel generator at hour t
$\rho_{T}$	Temperature coefficient	$C_{DIG,env}(t)$	Environmental cost of diesel generator at hour t
$T_{cell}$	Cell temperature	$C_{ESS}$	Total cost of ESS
$\sigma_{f}$	Total uncertainty in the objective function	$C_{ESS,cap}$	Capital cost of ESS
$x_{i}$	Uncertain parameters	$P_{grid}(t)$	Power purchased from the grid at hour t (kW)
$\sigma_{x_{i}}$	Standard deviation of uncertain parameter $x_{i}$	${Price}_{grid}(t)$	Grid electricity price at hour t ($/kWh)
$\frac{\partial_{f}}{\partial_{x_{i}}}$	Sensitivity of the cost function to	$E_{{CO}_{2}}$	CO2 emission coefficient
$W_{i}$	Weight of uncertain parameter${\;x}_{i}$	$E_{{SO}_{2}}$	SO2 emission coefficient
$\rho_{ij}$	Correlation coefficient between $x_{i}$ and $x_{j}$	$E_{{NO}_{x}}$	NOx emission coefficient
$F_{i}$	Total Expected Energy Not Supplied (kWh)	k	Shape parameter of Weibull distribution for wind speed
${EENS}_{G}$	EENS due to generation failures (kWh)	λ	Scale parameter of Weibull distribution for wind speed
$C_{ESS,O\&M}$	Operation and maintenance cost of ESS	γ	Coefficient specific to the wind turbine
${PGU}_{Costs}$	Costs of power generation units	β	Parameter of Beta distribution for PV power
SOC_ESS	State of charge of energy storage system	σ	Standard deviation of Normal distribution for loads
DG_Limits	Limits of distributed generation resources	T	Consumption threshold
$G_{r}$	Annual interest rate for cost calculations	B	Number of buses
$f_{1},f_{2}$	System performance metrics for optimization	t	set of hours
${Price}_{{CO}_{2}}$	Price of CO2 emissions	I	set of bus
${Price}_{{SO}_{2}}$	Price of SO2 emissions	α	Coefficient specific to the wind turbine

## Supporting information

S1 File Data. (DOCX)
