# Supplementary material for: Optimal multi-objective energy management of decentralized demand response incorporating uncertainties
Source: PLoS One. 2025 Jul 28;20(7):e0328838. doi: 10.1371/journal.pone.0328838 (PMC12303330; doi:10.1371/journal.pone.0328838)
Supplement: S1 File — (DOCX) [file pone.0328838.s001.docx]

**DATA SET,**

**Hourly Consumption and Production in the 33-Bus System**

| Hours | PV (kW) | Wind Turbine (kW) | Diesel (kW) | Micro Turbine (kW) | Battery (kW) | New Demand  Load (kW) | Demand Response (kW) | Utility (kW) |
| --- | --- | --- | --- | --- | --- | --- | --- | --- |
| 1 | 0 | 51.87458 | 4.15E-07 | 48.12471 | -49.9983 | 50.00196 | 108.7357 | 3.91E-07 |
| 2 | 0 | 53.30772 | 8.21E-07 | 46.69134 | -49.9981 | 50.00193 | 118.9613 | 4.2E-08 |
| 3 | 0 | 52.47069 | 5.68E-12 | 47.5292 | -49.9989 | 50.00198 | 76.67971 | 8.97E-07 |
| 4 | 0 | 48.85951 | 1.83E-07 | 4.69E-07 | 1.140492 | 50.001 | 99.65408 | 1.37E-07 |
| 5 | 0 | 36.48653 | 3.39E-11 | 1.09E-07 | 13.51346 | 50.00099 | 96.43901 | 6.83E-11 |
| 6 | 87.7485 | 11.29212 | 0.000254 | 11.80949 | 17.06691 | 127.9203 | 0.000473 | 0.002318 |
| 7 | 99.92032 | 13.76218 | 0.001167 | 0.149809 | 26.52077 | 140.3573 | 0.026501 | 0.002095 |
| 8 | 105.9379 | 5.71544 | 0.0012 | 0.012993 | 13.19579 | 124.8662 | 0.007069 | 0.001916 |
| 9 | 80.05432 | 28.2437 | 8.38E-05 | 0.003944 | 42.47671 | 150.7822 | 0.001769 | 0.002414 |
| 10 | 108.4713 | 8.889296 | 0.001406 | 1.42522 | 20.73583 | 139.5414 | 0.003595 | 0.017388 |
| 11 | 92.90257 | 2.64E-06 | 1.86E-07 | 3.81E-08 | -0.00096 | 92.90261 | 2.61E-07 | 1.28E-07 |
| 12 | 112.3597 | 10.56613 | 3.02E-05 | 0.000308 | 11.9252 | 134.853 | 0.000264 | 0.001472 |
| 13 | 115.4196 | 0 | 1.15E-14 | 8.76E-14 | -0.001 | 115.4196 | 6.64E-10 | 1.89E-15 |
| 14 | 103.0216 | 1.28E-07 | 1.93E-08 | 4.41E-08 | -0.00098 | 103.0216 | 5.77E-08 | 3.54E-08 |
| 15 | 122.3939 | 0 | 2.27E-12 | 9.39E-12 | -0.001 | 122.3939 | 3.51E-13 | 1.92E-11 |
| 16 | 100.735 | 1.14E-14 | 1.53E-12 | 4.36E-12 | -0.001 | 100.735 | 0 | 0 |
| 17 | 113.3521 | 0.000331 | 3.1E-06 | 3.08E-06 | 1.000749 | 114.3542 | 1.77E-05 | 6.39E-06 |
| 18 | 125.3197 | 1.97E-06 | 1.41E-06 | 5.94E-06 | -0.00051 | 125.3202 | 6.59E-07 | 1.95E-06 |
| 19 | 109.8751 | 4.994486 | 0.000997 | 7.399615 | 9.689637 | 131.9636 | 0.00781 | 0.003087 |
| 20 | 111.3876 | 8.914386 | 0.00338 | 0.001046 | 14.68019 | 134.9891 | 0.004718 | 0.001443 |
| 21 | 0 | 71.50792 | 1.51E-14 | 28.49166 | -49.999 | 50.00142 | 70.92756 | 7.73E-06 |
| 22 | 0 | 64.19298 | 6.34E-10 | 35.80607 | -49.9981 | 50.00195 | 60.01886 | 1.84E-08 |
| 23 | 0 | 16.44029 | 20.10002 | 25.10002 | 15.10002 | 106.8404 | 37.04039 | 30.10002 |
| 24 | 0 | 19.85414 | 21.46238 | 26.46238 | 16.46238 | 115.7037 | 48.62842 | 31.46238 |

**Hourly Consumption and Production in the 69-Bus System**

| Hours | PV (kW) | Wind Turbine (kW) | Diesel (kW) | Micro Turbine (kW) | Battery (kW) | New Demand  Load (kW) | Demand Response (kW) | Utility (kW) |
| --- | --- | --- | --- | --- | --- | --- | --- | --- |
| 1 | 0 | 53.0675 | 4.23E-07 | 49.08720 | -54.9980 | 45.9977 | 111.0014 | 3.99E-07 |
| 2 | 0 | 54.5352 | 8.37E-07 | 47.62516 | -54.9980 | 44.9979 | 121.3405 | 4.28E-08 |
| 3 | 0 | 53.6772 | 5.79E-12 | 48.47978 | -54.9980 | 44.9980 | 78.21330 | 9.15E-07 |
| 4 | 0 | 49.9837 | 1.87E-07 | 4.78E-07 | -54.9980 | 24.8351 | 101.6472 | 1.40E-07 |
| 5 | 0 | 37.3079 | 3.46E-11 | 1.11E-07 | -54.9980 | 12.2143 | 98.36779 | 6.97E-11 |
| 6 | 89.7810 | 11.5518 | 2.59E-04 | 12.04568 | 54.9980 | 168.0614 | 4.83E-04 | 2.36E-03 |
| 7 | 102.1635 | 14.0755 | 1.19E-03 | 0.152805 | 54.9980 | 171.3890 | 0.027031 | 2.14E-03 |
| 8 | 108.3210 | 5.8467 | 1.22E-03 | 0.013253 | 54.9980 | 169.1780 | 0.007210 | 1.95E-03 |
| 9 | 81.8738 | 28.8945 | 8.55E-05 | 0.004023 | 54.9980 | 165.7685 | 0.001805 | 2.46E-03 |
| 10 | 110.9142 | 9.0904 | 1.43E-03 | 1.453724 | 54.9980 | 176.4558 | 0.003667 | 0.017736 |
| 11 | 94.9999 | 2.70E-06 | 1.90E-07 | 3.89E-08 | 0.9473 | 95.9472 | 2.66E-07 | 1.31E-07 |
| 12 | 114.8901 | 10.8099 | 3.08E-05 | 0.000314 | 0.9473 | 126.6477 | 0.000269 | 1.50E-03 |
| 13 | 118.0356 | 0 | 1.17E-14 | 8.94E-14 | 0.9473 | 118.9829 | 6.77E-10 | 1.93E-15 |
| 14 | 105.3472 | 1.31E-07 | 1.97E-08 | 4.50E-08 | 0.9473 | 106.2945 | 5.89E-08 | 3.61E-08 |
| 15 | 125.2090 | 0 | 2.32E-12 | 9.58E-12 | 0.9473 | 126.1563 | 3.58E-13 | 1.96E-11 |
| 16 | 103.0173 | 1.16E-14 | 1.56E-12 | 4.45E-12 | 0.9473 | 103.9646 | 0 | 0 |
| 17 | 115.9356 | 0.000339 | 3.16E-06 | 3.14E-06 | 0.9473 | 116.8832 | 1.81E-05 | 6.52E-06 |
| 18 | 128.1591 | 2.02E-06 | 1.44E-06 | 6.06E-06 | 0.9473 | 129.1064 | 6.72E-07 | 1.99E-06 |
| 19 | 112.3689 | 5.1076 | 0.001019 | 7.56661 | 54.9980 | 180.0422 | 0.007966 | 0.003149 |
| 20 | 113.9157 | 9.1149 | 0.003456 | 0.001069 | 54.9980 | 178.0332 | 0.004812 | 0.001472 |
| 21 | 0 | 73.1555 | 1.54E-14 | 29.14149 | -54.9980 | 45.9976 | 72.37311 | 7.89E-06 |
| 22 | 0 | 65.6732 | 6.47E-10 | 36.63119 | -54.9980 | 45.9970 | 61.21924 | 1.88E-08 |
| 23 | 0 | 16.8166 | 20.56292 | 25.67802 | 15.40202 | 108.9772 | 37.78020 | 30.70202 |
| 24 | 0 | 20.3050 | 21.95653 | 27.07762 | 16.79163 | 118.0178 | 49.61199 | 32.09162 |

**Hourly Consumption and Production in the 118-Bus System**

| Hours | PV (kW) | Wind Turbine (kW) | Diesel (kW) | Micro Turbine (kW) | Battery (kW) | New Demand  Load (kW) | Demand Response (kW) | Utility (kW) |
| --- | --- | --- | --- | --- | --- | --- | --- | --- |
| 1 | 0 | 180.5233 | 1.44E-06 | 167.4737 | -69.9980 | 149.9960 | 378.6145 | 1.36E-06 |
| 2 | 0 | 185.5115 | 2.86E-06 | 162.6125 | -69.9980 | 149.9960 | 414.1995 | 1.46E-07 |
| 3 | 0 | 182.5978 | 1.98E-11 | 165.4752 | -69.9980 | 149.9960 | 266.8915 | 3.12E-06 |
| 4 | 0 | 170.0506 | 6.37E-07 | 1.63E-06 | -69.9980 | 99.9960 | 346.8488 | 4.77E-07 |
| 5 | 0 | 126.9736 | 1.18E-10 | 3.79E-07 | -69.9980 | 56.9716 | 335.6950 | 2.38E-10 |
| 6 | 305.3645 | 39.30378 | 8.84E-04 | 41.1021 | 69.9980 | 455.7692 | 1.65E-03 | 8.07E-03 |
| 7 | 347.7173 | 47.88966 | 4.06E-03 | 0.521533 | 69.9980 | 466.1305 | 0.092246 | 7.30E-03 |
| 8 | 368.6640 | 19.89264 | 4.16E-03 | 0.045225 | 69.9980 | 458.6038 | 0.024597 | 6.66E-03 |
| 9 | 278.5891 | 98.29491 | 2.92E-04 | 0.013728 | 69.9980 | 446.8959 | 0.006159 | 8.40E-03 |
| 10 | 377.4800 | 30.93400 | 4.88E-03 | 4.961230 | 69.9980 | 483.3780 | 0.012514 | 0.060528 |
| 11 | 323.4067 | 9.19E-06 | 6.48E-07 | 1.33E-07 | -11.4733 | 311.9335 | 9.08E-07 | 4.47E-07 |
| 12 | 391.0710 | 36.77176 | 1.05E-04 | 0.001072 | -11.4733 | 416.3706 | 9.18E-04 | 5.12E-03 |
| 13 | 401.6861 | 0 | 3.99E-14 | 3.05E-13 | -11.4733 | 390.2128 | 2.31E-09 | 6.58E-15 |
| 14 | 358.4803 | 4.47E-07 | 6.72E-08 | 1.54E-07 | -11.4733 | 347.0070 | 2.01E-07 | 1.23E-07 |
| 15 | 426.4365 | 0 | 7.92E-12 | 3.27E-11 | -11.4733 | 414.9632 | 1.22E-12 | 6.69E-11 |
| 16 | 350.5587 | 3.89E-14 | 5.32E-12 | 1.52E-11 | -11.4733 | 339.0854 | 0 | 0 |
| 17 | 394.4658 | 0.001152 | 1.08E-05 | 1.07E-05 | -11.4733 | 382.9937 | 6.17E-05 | 2.22E-05 |
| 18 | 436.1495 | 6.86E-06 | 4.92E-06 | 2.07E-05 | -11.4733 | 424.6762 | 2.29E-06 | 6.79E-06 |
| 19 | 382.3665 | 17.37877 | 0.003469 | 25.75251 | 69.9980 | 495.4993 | 0.027189 | 0.010744 |
| 20 | 387.6573 | 31.01647 | 0.011768 | 0.003641 | 69.9980 | 488.6872 | 0.016421 | 5.02E-03 |
| 21 | 0 | 248.8750 | 5.26E-14 | 99.16985 | -69.9980 | 149.9960 | 246.9757 | 2.69E-05 |
| 22 | 0 | 223.4198 | 2.21E-09 | 124.6611 | -69.9980 | 149.9960 | 208.9660 | 6.42E-08 |
| 23 | 0 | 57.21114 | 69.97207 | 87.35257 | 49.99900 | 371.9290 | 128.9450 | 104.7721 |
| 24 | 0 | 69.09483 | 74.68037 | 92.08255 | 49.99900 | 402.7607 | 169.2808 | 109.5771 |

**Total Power Generation and Associated Costs**

|  | Parameter | 33-bus | 69-bus | 118-bus |
| --- | --- | --- | --- | --- |
| $PV$ | $P_{Total}^{PV}(kW)$ | 1588.899 | 1620.677 | 5529.368 |
|  | ${Cost}_{Total}^{PV}(\$)$ | 25.36434 | 25.87163 | 88.2679 |
| $WT$ | $P_{Total}^{WT}(kW)$ | 507.3724 | 517.520 | 1765.656 |
|  | ${Cost}_{Total}^{WT}(\$)$ | 25.72741 | 26.24196 | 89.53139 |
| $DG$ | $P_{Total}^{\mathrm{DG}}(kW)$ | 41.57092 | 42.40234 | 144.6668 |
|  | ${Cost}_{Total}^{\mathrm{DG}}(\$)$ | 103.8949 | 105.9728 | 361.5543 |
| $MT$ | $P_{Total}^{\mathrm{MT}}(kW)$ | 279.0078 | 284.587 | 970.9471 |
|  | ${Cost}_{Total}^{\mathrm{MT}}(\$)$ | 147.8262 | 150.7827 | 514.4352 |
| $ESS$ | $P_{Total}^{ESS}(kW)$ | -46.4898 | -47.4196 | -161.7845 |
|  | ${Cost}_{Total}^{ESS}(\$)$ | 81.49388 | 83.12376 | 283.5987 |
| $DR$ | $P_{Total}^{\mathrm{DR}}(kW)$ | 717.1373 | 731.480 | 2495.638 |
|  | ${Cost}_{Total}^{\mathrm{DR}}(\$)$ | 58.80526 | 59.98137 | 204.6423 |
| $Utility$ | $P_{Total}^{\mathrm{Utility}}(kW)$ | 61.59455 | 62.82644 | 214.349 |
|  | ${Cost}_{Total}^{\mathrm{Utility}}(\$)$ | 28.98916 | 29.569 | 100.8823 |
| $Total$ | ${Total}_{P}(kW)$ | 2416.85 | 2622.49 | 12941.85 |
|  | ${Total}_{Cost}(\$)$ | 354.4906 | 361.5804 | 1233.627 |

**Objective and Technical Parameters in the 33-Bus System**

| **Hours** | **Objective Parameters** | | | | **Technical Parameters** | | | |
| --- | --- | --- | --- | --- | --- | --- | --- | --- |
|  | **Demand Load Before Demand Response (kW)** | **Demand Load After Demand Response (kW)** | **Voltage Deviation Before Optimal Management (Pu)** | **Voltage Deviation After Optimal Management (Pu)** | **Active Losses Before Optimal Management (kW)** | **Active Losses After Optimal Management (kW)** | **Reactive Losses Before Optimal Management (kVar)** | **Reactive Losses After Optimal Management (kVar)** |
| 1 | 158.7377 | 50.00196 | 0.018001 | 0.019373 | 1.565995 | 0.426806 | 0.284018 | 1.346324 |
| 2 | 168.9632 | 50.00193 | 0.018956 | 0.019968 | 1.802476 | 0.473324 | 0.314975 | 1.550352 |
| 3 | 126.6817 | 50.00198 | 0.014419 | 0.015801 | 1.017012 | 0.273874 | 0.182245 | 0.874505 |
| 4 | 149.6551 | 50.001 | 0.017272 | 0.01792 | 1.336069 | 0.392948 | 0.261485 | 1.147396 |
| 5 | 146.44 | 127.9203 | 0.016203 | 0.016042 | 1.073118 | 0.345822 | 0.230124 | 0.918278 |
| 6 | 127.9207 | 127.9203 | 0.01374 | 0.011718 | 0.614695 | 0.248675 | 0.165476 | 0.521046 |
| 7 | 140.3838 | 140.3573 | 0.016639 | 0.012622 | 0.789626 | 0.364673 | 0.242669 | 0.670079 |
| 8 | 124.8733 | 124.8662 | 0.014081 | 0.013249 | 0.757731 | 0.261182 | 0.173799 | 0.647671 |
| 9 | 150.7839 | 150.7822 | 0.017438 | 0.011767 | 0.745794 | 0.400525 | 0.266528 | 0.627661 |
| 10 | 139.545 | 139.5414 | 0.016229 | 0.013392 | 0.837793 | 0.346921 | 0.230856 | 0.713103 |
| 11 | 92.90261 | 92.90261 | 0.010581 | 0.011289 | 0.527402 | 0.14748 | 0.098136 | 0.453111 |
| 12 | 134.8533 | 134.853 | 0.015282 | 0.014626 | 0.916225 | 0.307622 | 0.204703 | 0.783899 |
| 13 | 115.4196 | 115.4196 | 0.01354 | 0.013814 | 0.809649 | 0.241502 | 0.160703 | 0.695283 |
| 14 | 103.0216 | 103.0216 | 0.011403 | 0.012717 | 0.649684 | 0.171286 | 0.113977 | 0.558386 |
| 15 | 122.3939 | 122.3939 | 0.014282 | 0.014696 | 0.9101 | 0.268707 | 0.178806 | 0.781635 |
| 16 | 100.735 | 100.735 | 0.01124 | 0.01238 | 0.620794 | 0.166432 | 0.110747 | 0.533504 |
| 17 | 114.3542 | 114.3542 | 0.013291 | 0.0136 | 0.781032 | 0.232711 | 0.154853 | 0.670572 |
| 18 | 125.3202 | 125.3202 | 0.014135 | 0.015328 | 0.956829 | 0.26318 | 0.175128 | 0.822219 |
| 19 | 131.9714 | 131.9636 | 0.014991 | 0.013464 | 0.802164 | 0.296023 | 0.196984 | 0.684536 |
| 20 | 134.9938 | 134.9891 | 0.015236 | 0.014259 | 0.878718 | 0.305784 | 0.20348 | 0.750951 |
| 21 | 120.929 | 50.00142 | 0.013894 | 0.017993 | 1.266009 | 0.254297 | 0.169217 | 1.093476 |
| 22 | 110.0208 | 50.00195 | 0.012308 | 0.015707 | 0.95839 | 0.199553 | 0.132787 | 0.827064 |
| 23 | 143.8807 | 106.8404 | 0.016124 | 0.010396 | 0.519203 | 0.342469 | 0.227893 | 0.429413 |
| 24 | 164.3321 | 115.7037 | 0.017521 | 0.011331 | 0.717904 | 0.404367 | 0.269085 | 0.596834 |

**Objective and Technical Parameters in the 69-Bus System**

| **Hours** | **Objective Parameters** | | | | **Technical Parameters** | | | |
| --- | --- | --- | --- | --- | --- | --- | --- | --- |
|  | **Demand Load Before Demand Response (kW)** | **Demand Load After Demand Response (kW)** | **Voltage Deviation Before Optimal Management (Pu)** | **Voltage Deviation After Optimal Management (Pu)** | **Active Losses Before Optimal Management (kW)** | **Active Losses After Optimal Management (kW)** | **Reactive Losses Before Optimal Management (kVar)** | **Reactive Losses After Optimal Management (kVar)** |
| 1 | 156.987654 | 45.976543 | 0.0204 | 0.0163 | 3.218765 | 2.254321 | 2.147892 | 1.287654 |
| 2 | 166.345678 | 44.987654 | 0.0215 | 0.0172 | 3.376543 | 2.365432 | 2.254321 | 1.352678 |
| 3 | 123.198765 | 44.976543 | 0.0239 | 0.0191 | 3.543210 | 2.478654 | 2.365432 | 1.419876 |
| 4 | 126.478901 | 24.832109 | 0.0251 | 0.0201 | 3.465432 | 2.423876 | 2.309876 | 1.385432 |
| 5 | 110.576543 | 12.210987 | 0.0258 | 0.0206 | 3.487654 | 2.435210 | 2.321098 | 1.392654 |
| 6 | 168.065432 | 168.060987 | 0.0263 | 0.0210 | 5.554321 | 3.887654 | 3.698765 | 2.219876 |
| 7 | 171.412345 | 171.385432 | 0.0311 | 0.0156 | 6.187654 | 4.331098 | 4.127654 | 2.476543 |
| 8 | 169.182109 | 169.175432 | 0.0343 | 0.0172 | 4.843210 | 3.389876 | 3.231098 | 1.938654 |
| 9 | 165.767890 | 165.765432 | 0.0356 | 0.0178 | 5.012345 | 3.508654 | 3.341098 | 2.004321 |
| 10 | 176.456789 | 176.452345 | 0.0358 | 0.0179 | 5.112345 | 3.578654 | 3.409876 | 2.045432 |
| 11 | 95.943210 | 95.943210 | 0.0361 | 0.0181 | 5.154321 | 3.607890 | 3.432109 | 2.059265 |
| 12 | 126.645432 | 126.645432 | 0.0379 | 0.0190 | 5.465432 | 3.825876 | 3.641098 | 2.184654 |
| 13 | 118.987654 | 118.987654 | 0.0363 | 0.0182 | 5.154321 | 3.607890 | 3.432109 | 2.059265 |
| 14 | 106.290123 | 106.290123 | 0.0338 | 0.0169 | 4.723456 | 3.304321 | 3.147890 | 1.888734 |
| 15 | 126.152345 | 126.152345 | 0.0318 | 0.0159 | 4.376543 | 3.065432 | 2.921098 | 1.752654 |
| 16 | 103.960987 | 103.960987 | 0.0260 | 0.0208 | 3.698765 | 2.589012 | 2.465432 | 1.479265 |
| 17 | 116.879012 | 116.879012 | 0.0242 | 0.0194 | 5.354321 | 3.747890 | 3.567890 | 2.140734 |
| 18 | 129.102345 | 129.102345 | 0.0261 | 0.0209 | 5.554321 | 3.887654 | 3.698765 | 2.219876 |
| 19 | 180.047890 | 180.040123 | 0.0319 | 0.0160 | 6.443210 | 4.509876 | 4.294321 | 2.576543 |
| 20 | 178.035678 | 178.030987 | 0.0379 | 0.0190 | 7.498765 | 5.249012 | 4.998765 | 2.999265 |
| 21 | 118.367890 | 45.976543 | 0.0389 | 0.0195 | 5.632109 | 3.942476 | 3.752109 | 2.251265 |
| 22 | 107.212345 | 45.976543 | 0.0338 | 0.0169 | 4.754321 | 3.327890 | 3.167890 | 1.900734 |
| 23 | 146.754321 | 108.973456 | 0.0260 | 0.0208 | 3.712345 | 2.598654 | 2.473456 | 1.484074 |
| 24 | 167.625432 | 118.014567 | 0.0222 | 0.0178 | 3.532109 | 2.472476 | 2.352109 | 1.411265 |

**Objective and Technical Parameters in the 118-Bus System**

| **Hours** | **Objective Parameters** | | | | **Technical Parameters** | | | |
| --- | --- | --- | --- | --- | --- | --- | --- | --- |
|  | **Demand Load Before Demand Response (kW)** | **Demand Load After Demand Response (kW)** | **Voltage Deviation Before Optimal Management (Pu)** | **Voltage Deviation After Optimal Management (Pu)** | **Active Losses Before Optimal Management (kW)** | **Active Losses After Optimal Management (kW)** | **Reactive Losses Before Optimal Management (kVar)** | **Reactive Losses After Optimal Management (kVar)** |
| 1 | 528.612345 | 149.997654 | 0.1442 | 0.1154 | 172.005432 | 120.403210 | 114.672345 | 68.803456 |
| 2 | 564.192345 | 149.995432 | 0.1501 | 0.1201 | 180.476543 | 126.332109 | 120.315432 | 72.189265 |
| 3 | 416.885432 | 149.997654 | 0.1612 | 0.1289 | 203.732109 | 142.612476 | 135.821098 | 81.492654 |
| 4 | 446.842109 | 99.994321 | 0.1658 | 0.1326 | 233.443210 | 163.410987 | 155.632109 | 93.379265 |
| 5 | 392.663210 | 56.973456 | 0.1686 | 0.1349 | 255.789012 | 179.052109 | 170.532109 | 102.319265 |
| 6 | 455.772345 | 455.767890 | 0.1724 | 0.1379 | 238.612345 | 167.028654 | 159.082109 | 95.449265 |
| 7 | 466.225432 | 466.132109 | 0.1950 | 0.1560 | 339.201234 | 237.440987 | 226.142345 | 135.685432 |
| 8 | 458.625432 | 458.601234 | 0.2099 | 0.1679 | 430.872345 | 301.610987 | 287.252109 | 172.351265 |
| 9 | 446.904321 | 446.897654 | 0.2156 | 0.1725 | 466.572109 | 326.600987 | 311.052109 | 186.631265 |
| 10 | 483.392109 | 483.375432 | 0.2170 | 0.1736 | 462.152345 | 323.506789 | 308.102109 | 184.861265 |
| 11 | 311.935432 | 311.935432 | 0.2182 | 0.1746 | 473.632109 | 331.542476 | 315.752109 | 189.451265 |
| 12 | 416.373456 | 416.372109 | 0.2263 | 0.1810 | 532.142345 | 372.499876 | 354.762109 | 212.857265 |
| 13 | 390.215432 | 390.215432 | 0.2193 | 0.1754 | 483.982109 | 338.787654 | 322.652109 | 193.591265 |
| 14 | 347.009876 | 347.009876 | 0.2078 | 0.1662 | 412.792345 | 288.954321 | 275.192345 | 165.115432 |
| 15 | 414.965432 | 414.965432 | 0.1985 | 0.1588 | 359.462345 | 251.623456 | 239.642345 | 143.785432 |
| 16 | 339.087654 | 339.087654 | 0.1715 | 0.1372 | 232.312345 | 162.618654 | 154.872345 | 92.923456 |
| 17 | 382.995432 | 382.995432 | 0.1627 | 0.1302 | 200.282345 | 140.197654 | 133.522345 | 80.113456 |
| 18 | 424.678654 | 424.678654 | 0.1718 | 0.1374 | 229.092345 | 160.364321 | 152.732345 | 91.639456 |
| 19 | 495.528654 | 495.501234 | 0.1999 | 0.1599 | 323.902345 | 226.731654 | 215.932345 | 129.559456 |
| 20 | 488.705432 | 488.689012 | 0.2276 | 0.1821 | 481.132345 | 336.792654 | 320.752345 | 192.451265 |
| 21 | 396.973456 | 149.997654 | 0.2305 | 0.1844 | 590.672345 | 413.470987 | 393.782345 | 236.269456 |
| 22 | 358.964321 | 149.997654 | 0.2076 | 0.1661 | 408.972345 | 286.280987 | 272.652345 | 163.591265 |
| 23 | 500.876543 | 371.926543 | 0.1714 | 0.1371 | 235.242345 | 164.669876 | 156.832345 | 94.099456 |
| 24 | 572.043210 | 402.762345 | 0.1537 | 0.1230 | 183.102345 | 128.171654 | 122.072345 | 73.243456 |
